# Silent cold-sensing neurons contribute to cold allodynia in neuropathic pain

**DOI:** 10.1093/brain/awab086

**Published:** 2021-03-09

**Authors:** Donald Iain MacDonald, Ana P Luiz, Federico Iseppon, Queensta Millet, Edward C Emery, John N Wood

**Affiliations:** Molecular Nociception Group, Wolfson Institute for Biomedical Research, University College London, London WC1E 6BT, UK

**Keywords:** pain, cold allodynia, neuropathic pain, potassium channels, sodium channels

## Abstract

Patients with neuropathic pain often experience innocuous cooling as excruciating pain. The cell and molecular basis of this cold allodynia is little understood. We used *in vivo* calcium imaging of sensory ganglia to investigate how the activity of peripheral cold-sensing neurons was altered in three mouse models of neuropathic pain: oxaliplatin-induced neuropathy, partial sciatic nerve ligation, and ciguatera poisoning. In control mice, cold-sensing neurons were few in number and small in size. In neuropathic animals with cold allodynia, a set of normally silent large diameter neurons became sensitive to cooling. Many of these silent cold-sensing neurons responded to noxious mechanical stimuli and expressed the nociceptor markers Na_v_1.8 and CGRPα. Ablating neurons expressing Na_v_1.8 resulted in diminished cold allodynia. The silent cold-sensing neurons could also be activated by cooling in control mice through blockade of K_v_1 voltage-gated potassium channels. Thus, silent cold-sensing neurons are unmasked in diverse neuropathic pain states and cold allodynia results from peripheral sensitization caused by altered nociceptor excitability.

## Teaser

MacDonald *et al.* show that cold allodynia—a hallmark of pain caused by cancer drugs like oxaliplatin, nerve injury or poisoning with ciguatoxin—leads to the activation of a set of large silent nociceptors in mice. Ablating these cells reverses cold allodynia, a phenomenon linked to K_v_1 channel activity in these cells.

## Introduction

Patients with chronic pain suffering from cold allodynia experience normally innocuous cooling as excruciating pain.^1^ Cold allodynia is a common symptom of neuropathic pain caused by chemotherapy, nerve injury or ciguatera poisoning, with a prevalence of up to 90%.^2^^,^^3^ How do neuropathic pain conditions with different aetiologies give rise to the same sensory disturbance of cold-evoked pain? Despite advances in our understanding of cold sensation, the pathophysiological mechanisms underlying cold allodynia remain elusive.

In the healthy state, peripheral sensory neurons show modality-specific responses to cold, with ‘labelled lines’ for both mild and extreme cooling.^4–9^ Cold detection involves cooling-gated ion channels like Trpm8, as well as sodium and potassium channels that control excitability at low temperatures.^10–17^ Mouse knockout studies suggest cold allodynia requires TRP channels and potassium channels expressed by unmyelinated C-fibres.^5^^,^^11^^,^^18–24^ A role for sodium channels enriched in A-fibres is also evident, however.^24–27^ Mechanistic investigation of the cells and molecules driving cold allodynia has proved difficult because of the challenge in recording large numbers of cold-responsive afferents, as well as the limitations of cold pain behaviour tests.^28^

To investigate if cold allodynia results from plasticity in peripheral sensory neurons, we used *in vivo* calcium imaging to explore how the activity of cold-sensing neurons is altered in neuropathic pain. Here we identify a previously undescribed set of large diameter silent cold-sensing neurons that contribute to cold allodynia in diverse neuropathic pain states and provide evidence for a potential role of K_v_1 potassium channels in unmasking their latent cold sensitivity.

## Materials and methods

### Animals

All animal procedures were approved by UCL ethical review committees and were discussed with Home Office inspectors to conform to UK Home Office regulations under Project licence P413329A2. The mouse lines used in this study are summarized in Supplementary Table 1.

All mouse lines were maintained on a C57/BL6 background and breeding strategies are as previously reported.^4^^,^^14^^,^^29–31^ Cre-dependent tdTomato reporter mice expressing GCaMP3 were generated by crossing subset-specific Cre mice with animals homozygous for Rosa-flox-stop tdTomato and homozygous for Pirt-GCaMP3. Na_v_1.8-Cre-dependent tdTomato and diphtheria toxin mice expressing GCaMP3 were generated by crossing Na_v_1.8 Cre mice with animals heterozygous for Rosa-flox-stop tdTomato, heterozygous Rosa-flox-stop DTA and homozygous for Pirt-GCaMP3.

Both male and female (> 6 weeks) mice were used for all experiments, and the number of mice of each sex used to generate each dataset is reported in Supplementary Table 2. The investigator was blinded to treatment and/or genotype. For genotyping, genomic DNA was isolated from ear biopsy for PCR. Genotyping primers are listed in Supplementary Table 3.

### Tamoxifen treatment

CGRPα-CreERT2 mice were given three 200 µl doses of a 1% tamoxifen solution on consecutive days between 6 and 8 weeks of age. TrkB-CreERT2 mice were given five 200 µl doses of a 1% tamoxifen solution on consecutive days between 8 and 10 weeks of age. Tamoxifen was made-up in 15% ethanol/85% sunflower oil.

### Neuropathic pain models

Standard behavioural tests were used to assess neuropathic pain and are described in detail in the Supplementary material. Animals were acclimatized to handling to minimize stress during the tests. All behavioural experiments were performed at a room temperature of 18–21°C.

#### Oxaliplatin

Chemotherapy-induced neuropathy was induced in mice by intraplantar injection of oxaliplatin into the left hind paw.^25^ Oxaliplatin was made up to a dose of 80 µg in 40 µl of 5% glucose solution, due to its instability in chloride-containing saline solution. Behaviour or imaging was assessed at least 3 h after injection.

#### Partial sciatic nerve injury

Peripheral nerve injury was studied in mice using a modified version of the Seltzer model.^32^ Surgical procedures were performed under isoflurane anaesthesia (2–3%). After the left thigh area was shaved and the skin sterilized with 70% ethanol, a longitudinal skin incision was made at the level of the femur. With the help of forceps, the muscle fibres were separated to allow visualization of the sciatic nerve. The partial nerve injury was induced by tying a tight ligature with a 6–0 silk suture around approximately one-third of the diameter of the sciatic nerve. The skin was then closed with a 6–0 Vicril suture and animals were kept in a warm enclosure until completely recovered. Behavioural testing was performed at 2 and 4 weeks post-surgery, and imaging experiments were carried out between 4 and 5 weeks after surgery.

#### Ciguatoxin-2

Ciguatoxin-2 (P-CTX-2) was a gift from Richard Lewis (University of Queensland). Because P-CTX-2 is highly lipophilic and sticks to plastic surfaces, it was made up to 10 µM in 50% methanol solution, stored in a glass vial at −20°C and aliquoted using a metal/glass Hamilton syringe. The stock solution was diluted in saline containing 1% bovine serum albumin (BSA) to produce a final concentration of 100 nM. Mice undergoing imaging or behavioural testing were given intraplantar injections with 20 µl of 100 nM P-CTX-2, and the effect of the drug was measured after 20–30 min.^24^

### *In vivo* calcium imaging

#### Acquisition

Mice expressing GCaMP3 were anaesthetized using ketamine (100 mg/kg), xylazine (15 mg/kg) and acepromazine (2.5 mg/kg). The depth of anaesthesia was confirmed by the pedal reflex and breathing rate. Animals were maintained at a constant body temperature of 37°C using a heated mat (VetTech). Lateral laminectomy was performed at spinal level L3–5. In brief, the skin was incised longitudinally, and the paravertebral muscles were cut to expose the vertebral column. Transverse and superior articular processes of the vertebra were removed using microdissection scissors and OmniDrill 35 (WPI). To obtain a clear image of the sensory neuron cell bodies in the ipsilateral dorsal root ganglion, the dura mater and the arachnoid membranes were carefully opened using microdissection forceps. The animal was mounted onto a custom-made clamp attached to the vertebral column (L1), rostral to the laminectomy. The trunk of the animal was slightly elevated to minimize interference caused by respiration. Artificial CSF [containing 120 mM NaCl, 3 mM KCl, 1.1 mM CaCl_2_, 10 mM glucose, 0.6 mM NaH_2_PO_4_, 0.8 mM MgSO_4_ and 1.8 mM NaHCO_3_ (pH 7.4) with NaOH] was perfused over the exposed dorsal root ganglion during the procedure to maintain tissue integrity, or the dorsal root ganglion was isolated by coating with silicone elastomer.

Images were acquired using a Leica SP8 confocal microscope. A 10× dry, 0.4-N.A. objective with 2.2 mm working distance was used with an image magnification of 0.75–3× optical zoom. GCaMP3 was excited using a 488 nm laser line (1–15% laser power). The reporter tdTomato was excited using a 552 nm laser line (1–15% laser power). Filtering and collection of the emission light was optimized to maximize yield and minimize cross-talk (Leica Dye 164 Finer, LasX software, Leica). GCaMP was detected using a hybrid detector (100% gain) and tdTomato using a photomultiplier tube (500–600 V gain). Pixel images (512 × 512) were captured at a frame rate of 1.55 Hz, bidirectional scan speed of 800 Hz and pixel dwell time of 2.44 µs.

Noxious and innocuous stimuli were applied to the left hind paw, ipsilateral to the exposed dorsal root ganglion. Thermal stimuli were applied by a Peltier-controlled thermode or by immersion of the paw in ice-water (nominally 0°C), acetone (100%) or water at 55°C using a Pasteur pipette. Mechanical stimuli were a noxious pinch with serrated forceps and innocuous brushing with a small paintbrush (ProArte-2) or cotton swab. An interval of at least 30 s separated each stimulus application. Pharmacological agents were delivered by intraplantar injection and are summarized in Supplementary Table 4.

#### Image analysis

Image stacks were registered to a reference frame using the FIJI plugin TurboReg (accurate rigid body transformation) to correct for *XY* drift. Stacks with excessive *Z* movement were excluded from the analysis. Regions of interest were manually drawn around responding cells using the free hand tool in FIJI. The time series of mean pixel intensity for each region of interest was extracted and smoothened by a four time-point moving average to remove high-frequency noise. Next, we calculated the derivative of the mean pixel intensity. Neurons were classed as responders if, within 30 s of stimulus application, the maximum derivative was greater than the baseline (10 s preceding stimulus application) derivative plus five standard deviations—that is, a *z*-score of at least 5. We then calculated the ΔF/F_0_ value for each response to obtain a normalized measure of change in fluorescence. Neurons that showed a ΔF/F_0_ < 0.25 were then discarded. Each trace was then manually screened as a further precaution against false positives. The remaining neurons that made up the responding population were then used for statistical analysis. The cross-sectional area for each region of interest in µm^2^ was also measured.

The red channel of the reference image was used to determine whether a cell was positive for tdTomato. Five regions of interest were drawn in the background areas of the image negative for tdTomato and the average pixel intensity measured to calculate the mean and standard deviation of the background red fluorescence. Red fluorescence in responding cells was *z*-scored versus the background value, and cells were counted as tdTomato positive if the *z*-score was >5.

### Quantification and statistical analysis

For the *in vivo* imaging experiments, *n* refers to the number of cells responding to any stimulus. For the electrophysiology experiments, *n* refers to the number of recorded cells. For all imaging and physiology data, the number of animals used is indicated in the legend. For the behavioural experiments, *n* refers to the number of animals.

Datasets are presented using appropriate summary statistics as indicated in the legend. For behavioural data, error bars denote the mean ± 95% confidence interval, unless otherwise indicated. For the *in vivo* imaging experiments, cells from all animals were pooled for analysis. These non-parametric data are summarized using medians with quartiles or cumulative probability plots. For the categorical data, 95% confidence intervals around proportions were estimated using the Wilson-Brown method.

The tests for the statistical comparison of each dataset are described in the figure legends. All test statistics are summarized in detail in Supplementary Table 5 for the main figures and Supplementary Table 6 for the Supplementary figures. When comparing two groups, the unpaired *t*-test or Mann-Whitney test was used. When comparing the distribution of cell cross-sectional areas for two groups, the Kolmogorov-Smirnov test was used. For more than two groups, the one-way ANOVA or Kruskall-Wallis test was used with *post hoc* tests corrected for multiple comparisons. When comparing the effect of two factors on multiple groups, a repeated-measures two-way ANOVA was used with *post hoc* tests corrected for multiple comparisons. For categorical data, proportions were compared using the χ^2^ test. Curve fitting was performed using linear regression or non-linear regression functions.

Statistical tests were all performed using GraphPad Prism 7. Differences were considered significant where *P *<* *0.05.

### Data availability

Data are available at https://doi.org/10.6084/m9.figshare.13293050.

## Results

### Silent cold-sensing neurons are unmasked during chemotherapy-induced neuropathy

**Figure 1:**
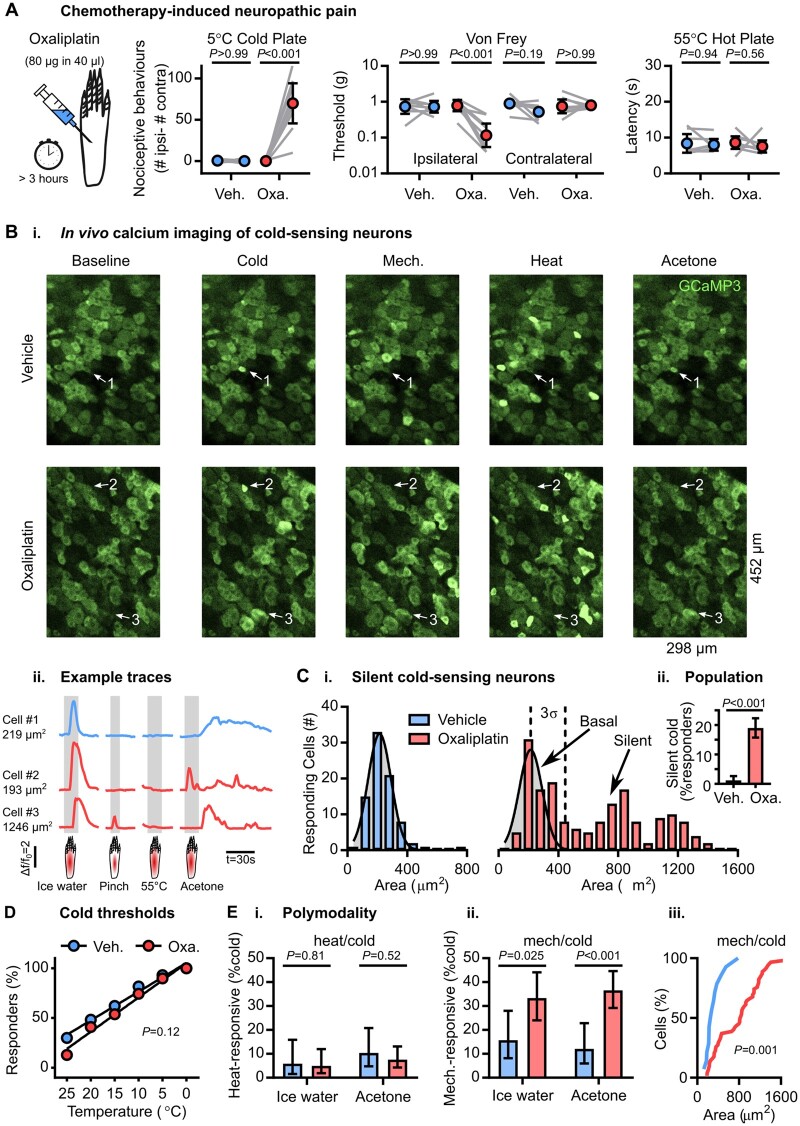
**Silent cold-sensing neurons are activated by oxaliplatin.** (**A**) Behavioural testing of the effect of intraplantar oxaliplatin injection on different sensory modalities (cold, mechanical and heat). *n* = 8 (five male and three female) for vehicle and *n* = 9 for oxaliplatin (five male and four female). Mean values before and after treatment were compared using repeated measures two-way ANOVA followed by *post hoc* Sidak’s test. Error bars denote 95% confidence interval. (**B**) Example images (**i**) and traces (**ii**) of cold-responding neurons in vehicle- and oxaliplatin-treated animals expressing GCaMP3. Cell 1 is a small diameter cold-sensing neuron in the vehicle condition, Cell 2 is a small diameter basal cold-sensing neuron after oxaliplatin and Cell 3 is a large diameter silent cold-sensing neuron unmasked by oxaliplatin that also responds to noxious mechanical stimuli. [**C**(**i**)] Histograms of the cross-sectional area of all neurons responding to any cold stimulus in the vehicle (*top*, blue, *n* = 82) and oxaliplatin (*bottom*, red, *n* = 179) groups. The distribution of areas for vehicle was fit by non-linear regression [least squares Gaussian; bin width is 80 µm^2^; mean = 214.9 µm^2^, SD (σ) = 77.29 µm^2^]. This model is plotted over the oxaliplatin data to aid comparison with the dashed line denoting 3 SD from the mean. The difference in the distribution of areas between groups was assessed by Kolmogorov-Smirnov test (*P* < 0.001). [**C**(**ii**)] Bar plot of the percentage of responding neurons classed as silent cold-sensing neurons in the vehicle and oxaliplatin groups. Proportions were compared using a χ^2^ test, and error bars denote 95% confidence intervals. (**D**) Relationship between the number of basal cold-sensing neurons and the drop in temperature can be fit by linear regression for both groups. For vehicle, *y* = −2.883*x* + 105.2, r^2^ = 0.9809, *n* = 87. For basal cold-sensing neurons after oxaliplatin, *y*  = −3.443*x* + 105, r^2^ = 0.9802, *n* = 39. The slopes are not significantly different (*P* = 0.12). (**E**) Quantification of the proportion of cold-sensing neurons responding to either heat (**i**) or mechanical (**ii**) stimuli in the vehicle and oxaliplatin groups. The proportion of polymodal neurons was compared using a χ^2^ test, and error bars denote confidence intervals. Ice water: *n*_veh_ = 51, *n*_oxa_ = 81. Acetone: *n*_veh_ = 58, *n*_oxa_ = 145. [**E**(**ii**)] Cumulative probability plots showing mechano-cold neurons tend to have larger cross-sectional areas in the oxaliplatin group, compared using the Kolmogorov-Smirnov test. *n*_veh_ = 14, *n*_oxa_ = 62. For this experiment, 383 neurons responding to any stimulus were recorded in eight vehicle-treated mice (five males and three females) and 542 cells were recorded from nine oxaliplatin-treated animals (five males and four females).

To investigate the mechanisms of cold allodynia, we used *in vivo* calcium imaging to explore how sensory neuron responses to cooling are altered during chemotherapy-induced neuropathy. Pirt-GCaMP3 mice expressing GCaMP3 in all sensory neurons were treated with oxaliplatin (80 µg/40 µl by hind paw intraplantar injection). Three hours after injection, both male and female mice displayed extreme cold hypersensitivity, as measured by the number of nociceptive and nocifensive behaviours when the animal was placed on a 5°C cold plate (Fig. 1A and Supplementary Fig. 1A). Oxaliplatin-treated mice also developed mechanical hypersensitivity but not heat hyperalgesia. The short-latency cold hypersensitivity observed after a single clinical dose of oxaliplatin (∼3 mg/kg) in this model mimics the rapid onset of cold allodynia in patients.^25^

Using laser-scanning confocal microscopy, we imaged cold-evoked calcium signals in sensory neuron somata of L4 dorsal root ganglia from oxaliplatin- and vehicle-treated animals. There was a dramatic change in the peripheral representation of cold following oxaliplatin treatment (Fig. 1B). In vehicle-treated mice, neurons responding to either ice-water or acetone were sparse and had small cross-sectional areas with a mean value of 214.9 µm^2^ [Fig. 1C(i)]. In oxaliplatin-treated animals, small cells still responded to cold; however, a novel, usually cold-insensitive population of large neurons also became activated by cooling. We consequently divided the cold-sensing neurons from the oxaliplatin-treated group into a basal population [within three standard deviations (SD) of the vehicle mean cross-sectional area] and an unmasked population (>3 SD away from this mean, >446.77 µm^2^) [Fig. 1C(i) and Supplementary Fig. 1B]. Because large neurons normally never respond to cooling but gain a *de novo* sensitivity to cold following oxaliplatin, we named them ‘silent cold-sensing neurons’. The percentage of cells classified as silent cold-sensing neurons rose from 1% (4/383) in vehicle- to 19% (102/542) in oxaliplatin-treated animals [Fig. 1C(ii)].

Interestingly, the response of many silent cold-sensing neurons to acetone continued for tens of seconds beyond the initial delivery of the stimulus (Fig. 1B). Consistent with this, oxaliplatin-treated animals showed prolonged nocifensive behaviour localized to the ipsilateral paw following acetone application (Supplementary Fig. 1C).

Cold allodynia could result from neurons that signal extreme cold becoming active at higher temperatures.^20^ However, oxaliplatin did not affect the thermal activation thresholds of basal cold-sensing neurons when the hind paw was stimulated with temperature drops delivered by a Peltier-controlled thermode (Fig. 1D). When we quantified the peak fluorescence intensity in response to cold as a surrogate for excitability, the cold-evoked fluorescence intensity in both the basal and silent populations in the oxaliplatin group was no different to the vehicle (Supplementary Fig. 1D). These data indicate oxaliplatin did not markedly affect the activation thresholds or excitability of basally-active cold-sensing neurons.

What effect did oxaliplatin have on neurons responding to other sensory modalities? The cross-sectional area of mechanically sensitive neurons was unchanged, but heat-activated cells showed a minor shift towards larger cells (Supplementary Fig. 1B). There was no increase in the proportion of heat- or mechanically sensitive cells (Supplementary Fig. 1E). The mechanical response magnitude was not altered, although the response to heat was reduced (Supplementary Fig. 1F). Thus, oxaliplatin treatment resulted in a modality-specific expansion in the peripheral representation of cold through the recruitment of silent cold-sensing neurons.

We previously showed that nociceptor polymodality is enhanced by inflammatory mediators.^4^ Interestingly, oxaliplatin increased the proportion of mechano-heat neurons from 19% (26/136) to 32% (61/193) (Supplementary Fig. 1G). However, few cold-sensing neurons responded to heat [Fig. 1E(i)]. By contrast, the fraction of cold-sensing neurons responding to noxious pinching was markedly increased [Fig. 1E(ii)]. For ice-water, this rose from 16% (8/51) to 33% (27/81) and for acetone from 12% (7/58) to 37% (53/145). This is likely to be an underestimate, because pinching targets a smaller receptive field than ice-water or acetone. These mechano-cold neurons were mainly large-diameter silent cold-sensing neurons [Fig. 1E(iii) and Supplementary Fig. 1H].

Importantly, silent cold-sensing neurons rarely responded to intermediate or low-threshold mechanical stimuli. Only 8% (15/199) of silent cold-sensing neurons responded to repeated stimulation with a 2 g Von Frey hair (Supplementary Fig. 2A and B). From 48 silent cold-sensing neurons tested, not one responded to stroking the glabrous or hairy skin using a paintbrush or cotton swab (Supplementary Fig. 2C and D). The mechanically sensitive subpopulation of silent cold-sensing neurons therefore primarily respond to high-threshold mechanical stimuli, consistent with their functional identity as nociceptors.

### Silent cold-sensing neurons are unmasked during peripheral nerve injury

**Figure 2:**
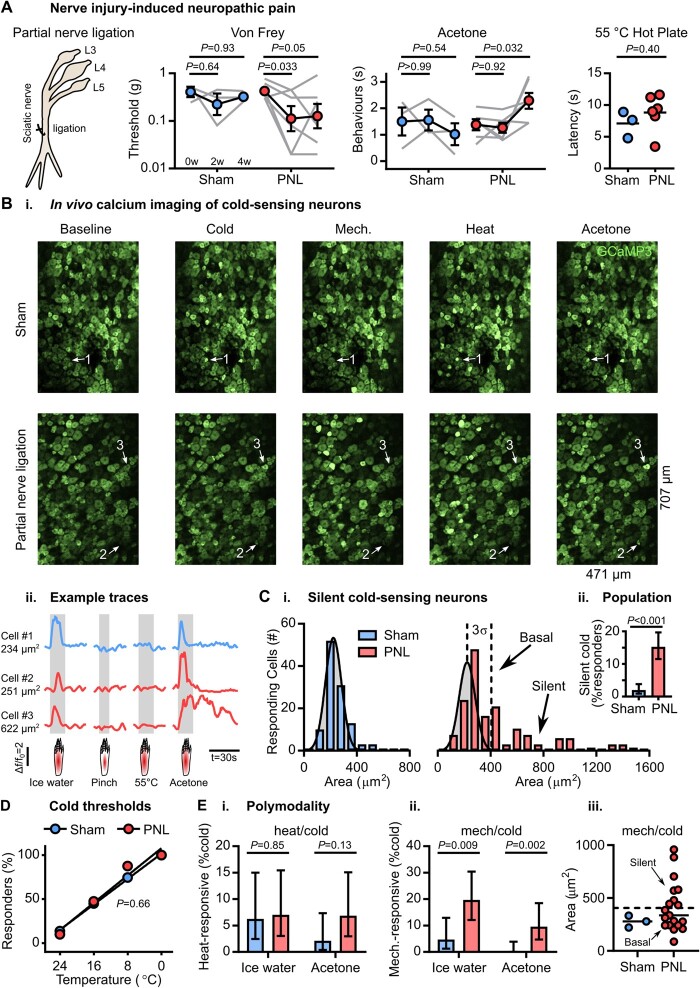
**Silent cold sensing neurons are activated after partial sciatic nerve ligation.** (**A**) Behavioural testing of the effect of PNL on different sensory modalities. *n* = 3 (one male and two females) for sham and *n* = 6 (three males and three females) for PNL. For Von Frey and acetone tests, means over time were compared using repeated measures two-way ANOVA followed by *post* *hoc* Sidak’s test. Hot plate latencies at 4 weeks were compared using unpaired *t*-test. Error bars denote the SEM. (**B**) Example images (**i**) and traces (**ii**) of cold-responding neurons in sham- and PNL-operated animals expressing GCaMP3. Cell 1 is a small diameter cold-sensing neuron in the sham condition; Cell 2 is a small diameter basal cold-sensing neuron after PNL; and Cell 3 is a large diameter silent cold-sensing neuron unmasked by PNL. [**C**(**i**)] Histograms of the cross-sectional area of all neurons responding to any cold stimulus in the sham (*top*, blue, *n* = 113) and PNL (*bottom*, red, *n* = 109) groups. The distribution of areas for sham was fit by non-linear regression (least squares Gaussian; bin width is 80 µm^2^; mean = 222.7 µm^2^, SD 60.9 µm^2^). This model is plotted over the PNL data to aid comparison with the dashed line denoting 3 SD from the mean. The difference in the distribution of areas between groups was assessed by Kolmogorov-Smirnov test (*P* < 0.001). [**C**(**ii**)] Bar plot of the percentage of responding neurons classed as silent cold-sensing neurons in the sham and PNL groups. Proportions were compared using a χ^2^ test, and error bars denote confidence intervals. (**D**) Relationship between the number of basal cold-sensing neurons and the drop in temperature can be fit by linear regression for both groups. For sham, *y* = −3.603*x* + 101.6, r^2^ = 0.9979, *n* = 51. For PNL, *y* = −3.875*x* + 107.8, r^2^ = 0.9598, *n* = 40. The slopes are not significantly different (*P* = 0.66). (**E**) Quantification of the proportion of cold-sensing neurons responding to either heat (**i**) or mechanical (**ii**) stimuli in the sham and PNL groups. The proportion of polymodal neurons was compared using a χ^2^ test, and error bars denote confidence intervals. Ice-water: *n*_sham_ = 64, *n*_PNL_ = 71. Acetone: *n*_sham_ = 95, *n*_PNL_ = 73. [**E**(**iii**)] Scatter plots showing mechano-cold neurons have both small and large cross-sectional areas in the PNL group. *n*_sham_ = 3, *n*_PNL_ = 19. For this experiment, 373 neurons responding to any stimulus were recorded in three sham-operated mice (one male and two females) and 297 cells were recorded from six PNL-operated animals (three males and three females).

Are silent cold-sensing neurons unmasked in other neuropathies? To mimic nerve injury-induced neuropathic pain, we performed partial sciatic nerve ligation (PNL) on Pirt-GCaMP3 mice. Two weeks after surgery, nerve injured animals developed mechanical, but not cold, hypersensitivity. At 4 weeks, we observed a modest cold hypersensitivity using both the acetone and unilateral cold plate test but no difference in heat nociception. (Fig. 2A and Supplementary Fig. 3A). Qualitatively similar results were obtained in both males and females for all assays (Supplementary Fig. 3B).

We therefore imaged both nerve-injured and sham-operated mice between 4 and 5 weeks post-surgery (Fig. 2B). In sham-operated mice, cold-sensing neurons were small in size with a mean area of 222.7 µm^2^ [Fig. 2C(i)]. After nerve injury, normally cold-insensitive, large-diameter neurons responded to both ice-water and acetone stimuli [Fig. 2C(i) and Supplementary Fig. 3C]. Neurons with cross-sectional areas >3 SD away from the sham mean (>405.4 µm^2^) were classified as silent cold-sensing neurons. The silent cold-sensing neuron population expanded from 2% (7/373) to 15% (45/291) [Fig. 2C(ii)]. Fewer silent cold-sensing neurons were recruited by nerve injury than oxaliplatin, consistent with a less profound behavioural cold hypersensitivity.

Nerve injury did not alter the thermal activation thresholds of basal cold-sensing neurons (Fig. 2D), but the effect on excitability was complex. Acetone-evoked activity was enhanced in the basal population, while silent cells showed reduced responses to ice-water (Supplementary Fig. 3D). The number of heat-cold polymodal neurons was unchanged [Fig. 2E(i)]; however the proportion of mechano-cold cells was significantly increased [Fig. 2E(ii)]. For ice-water, this rose from 5% (3/64) to 20% (14/71) and for acetone from 0% (0/95) to 10% (7/73). Mechano-cold cells comprised both basal and silent cold-sensing neurons, based on cross-sectional area [Fig. 2E(iii) and Supplementary Fig. 3E]. In addition, when the glabrous skin was lightly stroked with a paintbrush, just 2% (1/41) of silent cold-sensing neurons unmasked by nerve injury responded to this low threshold mechanical stimulus (Supplementary Fig. 2E). Nerve injury and oxaliplatin thus have broadly similar effects on the peripheral representation of cold, unmasking silent cold-sensing neurons that also sometimes respond to noxious mechanical stimuli.

Neurons responding to other modalities were variably affected by nerve injury. There was no change in cell area for mechanical stimuli, but heat-activated neurons were larger (Supplementary Fig. 3C). Significantly more neurons responded to pinch (38% versus 28%), and there was a trend towards fewer responses to heat (40% versus 47%) (Supplementary Fig. 3F). We saw no difference in the intensity of the response to noxious heat, but pinch-evoked peak activity was decreased (Supplementary Fig. 3G). Unlike oxaliplatin, there was no enhancement of mechano-heat polymodality (Supplementary Fig. 3H).

### Silent cold-sensing neurons are unmasked during ciguatera poisoning

**Figure 3:**
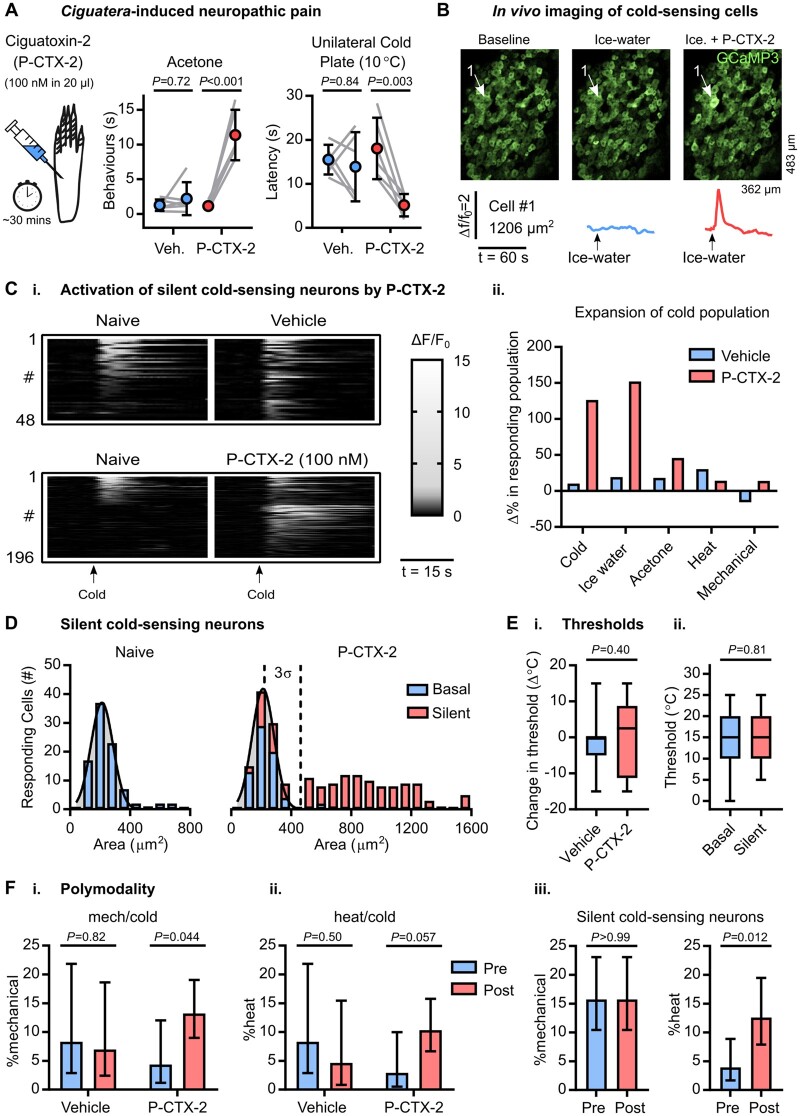
**Silent cold-sensing neurons are activated by ciguatoxin-2.** (**A**) Behavioural testing of the effect of intraplantar injection of 100 nM ciguatoxin-2 (P-CTX-2) on cold sensitivity *n* = 6 for sham vehicle (three males and three females) and *n* = 6 for P-CTX-2 (three males and three females). Means were compared by repeated measures two-way ANOVA followed by *post* *hoc* Sidak’s test. Error bars denote 95% confidence interval. (**B**) Example images and traces of a large-diameter neuron (Cell 1) that is basally cold-insensitive but begins to respond to cooling after treatment with P-CTX-2. [**C**(**i**)] Heat map showing the effect of P-CTX-2 on the number of neurons responding to a cold ice-water stimulus. *n* = 48 for vehicle and *n* = 196 for P-CTX-2. The bar corresponds to 15 s. [**C**(**ii**)] Summary of the change in the number of sensory neurons responding to each modality after treatment with P-CTX-2. (**D**) Histograms of cross-sectional area of all neurons responding to any cold stimulus in the naïve state (*left*, blue, *n* = 91) and after P-CTX-2 (*right*). For P-CTX-2, blue denotes basally responsive neurons that maintained their response to cold (*n* = 70) and red denotes the silent cold-sensing neurons that were unmasked after treatment (*n* = 136). The distribution of areas in the naïve state was fit by non-linear regression (least squares Gaussian; bin width is 80 µm^2^; mean = 212.4 µm^2^, SD 73.33 µm^2^). This model is plotted over the P-CTX-2 data to aid comparison with the dashed line denoting 3 SD from the mean. The different in the distribution of areas between groups was assessed by Kolmogorov-Smirnov test (*P* < 0.001). [**E**(**i**)] Box plot of the change in activation threshold of basally cold-sensitive neurons before and after treatment with vehicle (*n* = 35) or P-CTX (*n* = 8). [**E**(**ii**)] Box plot of the thermal activation threshold of all silent cold-sensing neurons unmasked by P-CTX-2 (*n* = 43) compared to all cold-sensing neurons recorded from naïve mice (*n*** **= 62). Medians were compared by Mann-Whitney test. (**F**) Quantification of the proportion of neurons responding ice-water that were also sensitive to either mechanical (**i**) or heat (**ii**) before and after treatment. Vehicle: *n*_pre_ = 36, *n*_post_ = 43. P-CTX-2: *n*_pre_ = 69, *n*_post_ = 174. [**F**(**iii**)] Comparison of the proportion of silent cold-sensing neurons that were responsive to other modalities before and after the induction of cold-sensitivity by P-CTX-2. *n* = 127. The proportion of polymodal neurons was compared using a χ^2^ test, and error bars denote 95% confidence intervals. For this experiment, 615 neurons responding to any stimulus either before or after treatment were recorded in 10 P-CTX-2-injected mice (four males and six females) and 193 cells were recorded from three vehicle-injected animals (two males and one female).

Both oxaliplatin and nerve injury show a delayed onset of cold hypersensitivity. As our imaging preparation was terminal, we could not follow mice in real-time to determine if silent cold-sensing neurons are truly silent in the naive state. To induce cold allodynia within the same imaging session, we turned to a mouse model of ciguatera poisoning, a marine toxin-induced neuropathy characterized by cold pain in the extremities that results from consuming contaminated seafood.^24^ Hind paw intraplantar injection of ciguatoxin-2 (P-CTX-2, 100 nM) evoked cold pain by 30 min in both male and female mice, as judged by the acetone and 10°C unilateral cold plate test (Fig. 3A and Supplementary Fig. 4A).

We therefore imaged sensory neuron cold sensitivity over the same time course (Fig. 3B). After 30 min, P-CTX-2 induced robust responses to cooling in numerous initially cold-insensitive cells [Fig. 3C(i)]. Some neurons actually lost their response to cold; however, this was counterbalanced by the large number of cells that gained *de novo* cold sensitivity, resulting in a net expansion of cold population, especially to ice-water. The number of neurons responsive to any cold stimulus rose from 91 to 206 and for ice water from 69 to 174. P-CTX-2 did not affect how many cells responded to other modalities [Fig. 3C(ii)]. Silent cold-sensing neurons unmasked by P-CTX-2 were also large, with a mean cross-sectional area of 820.1 µm^2^ (Fig. 3D). The size of heat-sensing cells was not markedly altered, although more small neurons responded to noxious pinch (Supplementary Fig. 4B).

How did P-CTX-2 treatment affect basally active cold cells? We quantified the change in the threshold of these cells after either P-CTX-2 or vehicle and saw no difference [Fig. 3E(i)]. Interestingly, P-CTX-2 reduced the peak cold response of these neurons compared to vehicle (Supplementary Fig. 4C). Silent cold-sensing neurons showed similar thermal activation thresholds to the basal population [Fig. 3E(ii)], and their activity was not greater (Supplementary Fig. 4D). P-CTX-2 did not affect the peak response to other modalities (Supplementary Fig. 4E); however, there was an increase in the fraction of polymodal mechano-heat neurons from 12–25% (Supplementary Fig. 4F).

P-CTX-2 increased mechano-cold polymodal neurons responding to ice-water from 4–13% [Fig. 3F(i)]. Heat/cold polymodality was also enhanced, albeit not significantly [Fig. 3F(ii)]. Interestingly, the proportion of identified silent cold-sensing neurons that responded to noxious mechanical stimuli was, at 16%, the same in the naïve state and after P-CTX-2. This indicated that at least some silent cold-sensing are responsive to noxious mechanical stimuli before the induction of neuropathy. Few heat/cold cells showed a basal response to heat, indicating heat sensitivity is conferred by P-CTX-2 [Fig. 3F(iii)]. Results were broadly similar when we looked only at the cold-sensing neurons defined by their response to acetone (Supplementary Fig. 4G). Finally, cold-sensing neurons unmasked by P-CTX-2 almost never responded to light touch stimuli, either before or after treatment (Supplementary Fig. 2F).

These findings demonstrate cold allodynia induced by P-CTX-2 involves unmasking silent cold-sensing neurons, some of which are pinch-activated mechano-nociceptors. Aetiologically distinct neuropathic pain states therefore give rise to cold pain by a similar mechanism of recruiting cold-insensitive sensory neurons to become cold-responsive.

### Molecular characterization of silent cold-sensing neurons that drive cold allodynia in neuropathic pain

**Figure 4:**
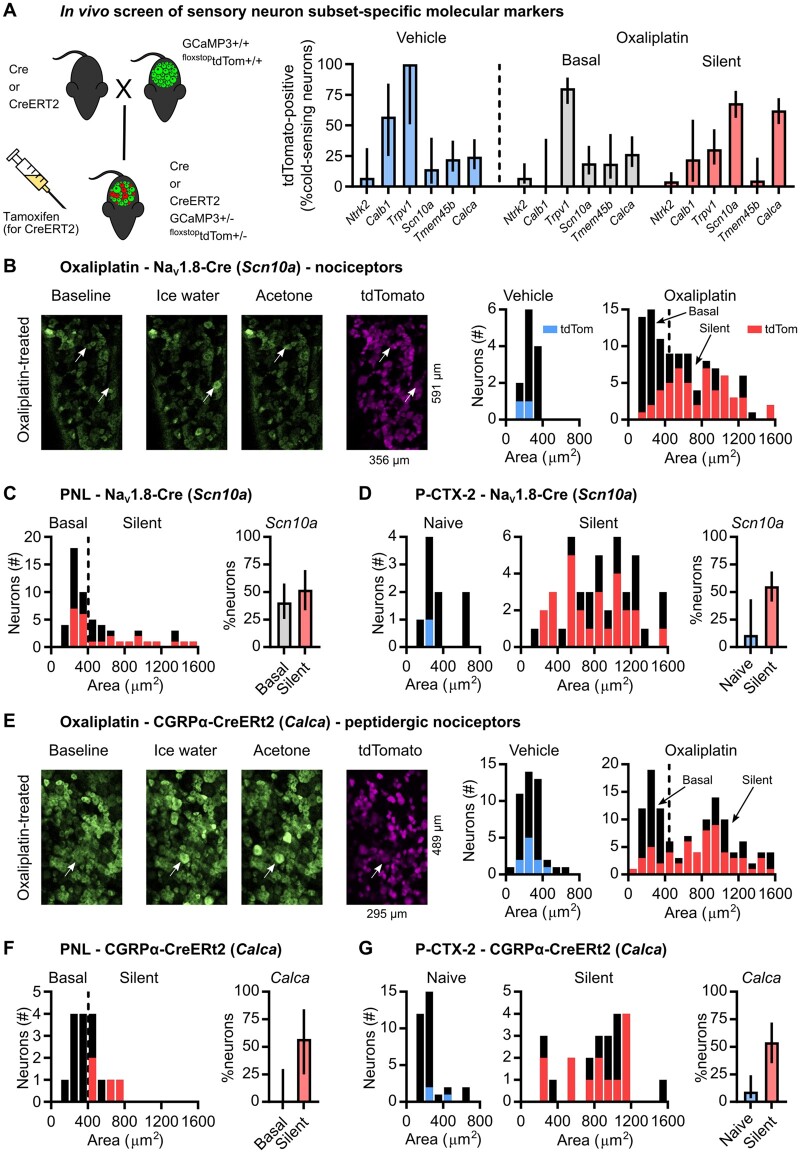
**Silent cold-sensing neurons express peptidergic nociceptor molecular markers Na_v_1.8 and CGRPα.** (**A**) Cartoon (*left*) of breeding strategy used to generate GCaMP3 reporter mice for each subset of interest. Bar plot (*right*) showing overlap of reporter expression for each marker with functionally defined cold-sensing neurons. TrkB-CreERT2 (*Ntrk2*): *n*_veh_ = 14 from two mice (one male and one female), *n*_oxa_ = 112 from three mice (two males and one female). Calb1-Cre (*Calb1*): *n*_veh_ = 7 from one mouse (one male), *n*_oxa_ = 15 from two mice (two females). Trpv1-Cre (*Trpv1*): *n*_veh_ = 4 from one mouse (one male), *n*_oxa_ = 87 from two mice (one male and one female). Na_v_1.8-Cre (*Scn10a*): *n*_veh_ = 14 from four mice (two males and two females), *n*_oxa_ = 108 from six mice (four males and two females). Tmem45b-Cre (*Tmem45b*): *n*_veh_ = 40 from one mouse (one male), *n*_oxa_ = 36 from three mice (two males and one female). CGRPα-CreERT2 (*Calca*): *n*_veh_ = 45 from three mice (one male and two females), *n*_oxa_ = 122 from two mice (one male and one female). (**B**) Example images (*left*) and histograms (*right*) showing overlap of Na_v_1.8-Cre-dependent tdTomato expression with cold-sensing neurons of different sizes in vehicle- and oxaliplatin-treated mice. Same data as in **A**. (**C**) Histogram (*left*) and bar plot (*right*) showing overlap of Na_v_1.8-Cre-dependent tdTomato expression with different types of cold-sensing neurons in PNL-operated mice. *n* = 57 cells from two mice (one male and one female). (**D**) Histograms (*left*) and bar plot (*right*) showing overlap of Na_v_1.8-Cre-dependent tdTomato expression with basally active and silent cold-sensing neurons in mice treated with P-CTX-2. *n* = 56 cells from four mice (one male and three females). (**E**) Example images (*left*) and histograms (*right*) showing overlap of CGRPα-CreERT2-dependent tdTomato expression with cold-sensing neurons of different sizes in vehicle- and oxaliplatin-treated mice. Same data as in **A**. (**F**) Histogram (*left*) and bar plot (*right*) showing overlap of CGRPα-CreERT2-dependent tdTomato expression with different types of cold-sensing neurons in PNL-operated mice. *n* = 16 cells from one mouse (one male). (**G**) Histograms (*left*) and bar plot (*right*) showing overlap of CGRPα-CreERT2-dependent tdTomato expression with basally active and silent cold-sensing neurons in mice treated with P-CTX-2. *n* = 56 cells from two mice (one male and one female). Error bars denote 95% confidence intervals. As these data were obtained as part of an exploratory screen, no statistical hypothesis testing was performed.

What is the molecular identity of silent cold-sensing neurons? We crossed subset-specific Cre or CreERT2 mice with animals harbouring a Cre-dependent tdTomato reporter on a Pirt-GCaMP3 background. This generated progeny expressing GCaMP3 in all sensory neurons but with tdTomato expression restricted to the cellular subset of interest (Fig. 4A). Consequently, we were able to ask if functionally identified silent cold-sensing neurons express molecular markers labelling major subpopulations of sensory neurons.^33^^,^^34^ We focused on oxaliplatin neuropathy because of its ease, reproducibility and clinical relevance. The percentage of neurons responding to any cold stimulus in vehicle- and oxaliplatin-treated mice expressing each molecular marker is summarized in Fig. 4A, split as before into basal and silent populations.

*Ntrk2* (TrkB-CreERT2) is a molecular marker for Aδ-fibre low threshold mechanoreceptors and was expressed by just 4% of silent cold-sensing neurons (Fig. 4A). Silent cold-sensing neurons also showed minimal expression of the Aβ-fibre low threshold mechanoreceptor marker *Calb*1 (Calb1-Cre) with only 22% overlap (Fig. 4A). The molecular profile therefore concurs with the functional data that silent cold-sensing neurons are unlikely to be low threshold mechanoreceptors, despite their large size. However, silent cold-sensing neurons showed only a 30% overlap with *Trpv1* lineage neurons, which encompass a broad mixture of nociceptors and thermosensors (Fig. 4A).

Given that some silent cold cells respond to noxious mechanical stimuli, we hypothesized that they express *Scn10a*, a mechano-nociceptor marker.^29^^,^^35^ We previously showed that under physiological conditions the bulk of neurons sensing cold down to 0°C are negative for *Scn10a*, which encodes sodium channel Na_v_1.8.^14^ In agreement with this, very few small-sized cold-sensing neurons were marked by Na_v_1.8-Cre in animals treated with either vehicle (14%) or oxaliplatin (19%). In contrast, we found 68% of the large diameter silent cold-sensing neurons unmasked by oxaliplatin expressed Na_v_1.8 (Fig. 4A and B). When we examined animals with cold allodynia evoked by PNL, 52% of silent cold-sensing neurons were marked by Na_v_1.8, although 41% of smaller cells also expressed Na_v_1.8 in this model (Fig. 4C). Similarly, 55% of the silent cold-sensing neurons unmasked by P-CTX-2 were positive for Na_v_1.8, with only 11% overlap among the cold cells active in the naïve state (Fig. 4D). Thus, in all three neuropathic pain models tested here, most silent cold-sensing neurons expressed Na_v_1.8, forming a unique subpopulation of large diameter *Scn10a*-expressing nociceptors.

To determine which nociceptor subset silent cold-sensing neurons belonged to, we used a Tmem45b-Cre mouse line to identify *Tmem45b*-expressing non-peptidergic nociceptors.^33^ Just 5% of silent cold cells in oxaliplatin neuropathy expressed Tmem45b (Fig. 4A). On the other hand, 62% of silent cold-sensing neurons in oxaliplatin neuropathy overlapped with *Calca*-expressing peptidergic nociceptors labelled with CGRPα-CreERT2 (Fig. 4A and E). The silent cold-sensing neurons also showed substantial overlap with *Calca*-expressing cells in mice with cold allodynia evoked by PNL (57%) or P-CTX-2 (54%), identifying silent cold cells as a set of peptidergic nociceptors commonly involved in cold allodynia (Fig. 4F and G). Hence, expression of *Scn10a* or *Calca* can be used to differentiate the silent and basal cold-sensing neurons.

Next, we restricted our analysis only to those silent cold-sensing neurons that were also sensitive to noxious mechanical stimuli and obtained a similar pattern of marker expression. In the oxaliplatin model, silent cold cells responsive to pinch were strongly positive for *Scn10a* (53%) and *Calca* (68%) but mainly negative for *Trpv1* (27%), with none expressing *Tmem45b*, *Calb1* or *Ntrk2* (Supplementary Fig. 5A). In P-CTX-2-treated mice, we also saw that the silent mechano-cold neurons largely expressed both *Scn10a* (54%) and *Calca* (83%) (Supplementary Fig. 5B). Thus, the mechanosensory subpopulation of silent cold-sensing neurons are also likely to be peptigdergic nociceptors.

**Figure 5:**
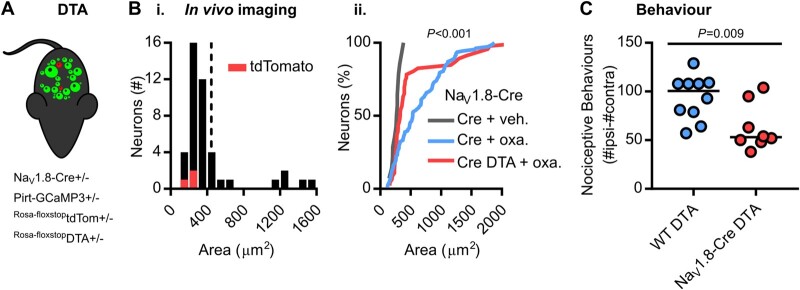
**Diphtheria toxin-mediated ablation of Na_v_1.8-positive nociceptors decreases oxaliplatin-induced cold allodynia.** (**A**) Cartoon of diphtheria toxin-mediated ablation of Na_v_1.8-positive neurons. [**B**(**i**)] Histogram of cross-sectional areas of all cold-sensing neurons imaged in Na_v_1.8-Cre DTA mice treated with oxaliplatin. [**B**(**ii**)] Cumulative probability plot of cell areas in oxaliplatin-treated Na_v_1.8-Cre (blue) and Na_v_1.8-Cre DTA (red) mice, compared by Kolmogorov-Smirnov test. The distribution of cell areas in vehicle-treated Na_v_1.8-Cre mice is shown for comparison. *n* = 108 cells from six oxaliplatin-treated Na_v_1.8-Cre mice (four males and two females), *n* = 46 cells from two oxaliplatin-treated Na_v_1.8-Cre DTA mice (one male and one female) and *n* = 14 cells from four vehicle-treated Na_v_1.8-Cre mice (two males and two females). (**C**) Quantification of the number of nociceptive behaviours in 5 min on the 5°C cold plate in 10 control and 8 Na_v_1.8-Cre DTA mice treated with oxaliplatin.

Finally, we used diphtheria toxin to conditionally ablate silent cold-sensing neurons marked by Na_v_1.8-Cre to test their causal role in cold allodynia (Fig. 5A). This ablation encompasses all cells expressing Na_v_1.8 and is not restricted to silent cold-sensing neurons. Imaging of mice where *Scn10a*-positive nociceptors are ablated showed that very few of the large-diameter silent cold-sensing neurons are unmasked by oxaliplatin compared to Na_v_1.8-Cre mice lacking DTA (Fig. 5B). The small basal cold-sensing neurons are retained after the killing of *Scn10a*-positive neurons. Although nocifensive behaviour was not fully abolished, we observed a ∼50% decrease in oxaliplatin-evoked cold hypersensitivity in Na_v_1.8-Cre DTA animals (Fig. 5C). The molecular identification and subsequent manipulation of silent cold-sensing neurons thus corroborates their causal contribution to cold allodynia in neuropathic pain.

### Molecular basis of cold detection by silent cold-sensing neurons

**Figure 6:**
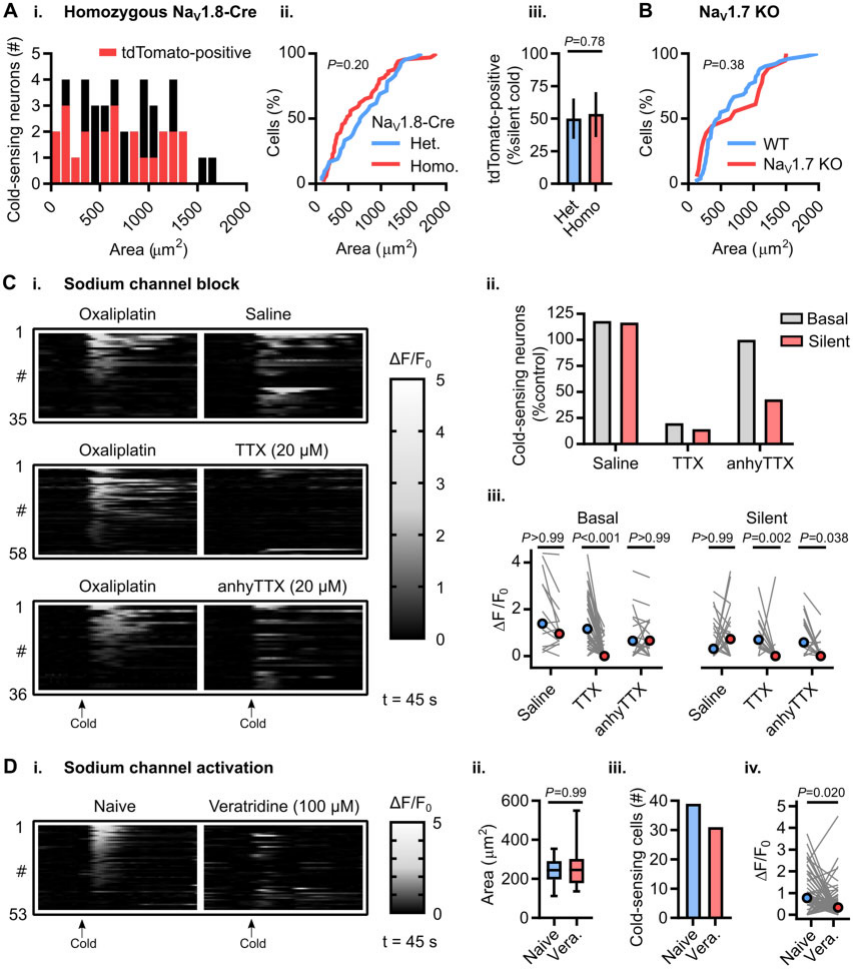
**Voltage-gated sodium channel Na_v_1.6 is required for excitability, but is not sufficient for cold sensitivity, of silent cold-sensing neurons.** [**A**(**i**)] Histogram of cross-sectional areas of all cold-sensing neurons imaged in homozygous Na_v_1.8-Cre tdTomato mice with oxaliplatin. [**A**(**ii**)] Cumulative probability plot of cell areas in oxaliplatin-treated heterozygous and homozygous Na_v_1.8-Cre mice, compared by Kolmogorov-Smirnov test. [**A**(**iii**)] Bar plot showing the proportion of silent cold-sensing neurons expressing tdTomato in heterozygous and homozygous Na_v_1.8-Cre mice, compared using a χ^2^ test. *n *= 66 cells from three heterozygous (two males and one female) and *n* = 42 cells from three homozygous (one male and two females) Na_v_1.8-Cre mice. (**B**) Cumulative probability plot of cell areas in oxaliplatin-treated wild-type and Na_v_1.7 KO mice, compared by Kolmogorov-Smirnov test. *n* = 51 cells from five wild-type (one male and four females) and *n* = 18 cells from two Na_v_1.7 knockout mice (two females). (**C**) Heat maps (**i**) and quantification (**ii**) showing the effect of intraplantar injection of different sodium channel blockers on the number of basal and silent cold-sensing neurons in mice pretreated with oxaliplatin. [**C**(**iii**)] Line plot showing the effect of blockers on median peak response, compared using Kruskall-Wallis test followed by Dunn’s multiple comparisons test. *n* = 35 cells from three mice for saline (one male and two females), *n *= 58 cells from four mice for TTX (three males and one female), *n* = 36 cells from two mice for 4,9-anhydrous-TTX (two females). [**D**(**i**)] Heat map showing the effect of sodium channel activation by intraplantar veratridine injection on the activity of cold-sensing neurons. [**D**(**ii**)] Box plot showing the size of cold-sensing cells is unaffected by veratridine, compared by Mann-Whitney test. [**D**(**iii**)] Bar plot showing veratridine reduces the number of cold-sensing neurons from 39 to 31. [**D**(**iv**)] Line plot showing veratridine reduces the response magnitude of cold-sensing cells, compared by Wilcoxon matched-pairs signed rank test. *n* = 53 cells from three mice (three males).

Which sodium channel isoform is required for silent cold-sensing neuron excitability? For mechanistic investigation, we focused on silent cold-sensing neurons responding to ice-water stimuli in the oxaliplatin model. Because Cre is knocked in directly at the Na_v_1.8 locus, homozygous Na_v_1.8-Cre mice lack both wild-type *Scn10a* alleles and are thus Na_v_1.8 nulls without Na_v_1.8-dependent tetrodotoxin (TTX)-resistant voltage-gated sodium currents (Supplementary Fig. 6B). When we treated homozygous Na_v_1.8-Cre mice expressing Cre-dependent tdTomato on a Pirt-GCaMP3 background with oxaliplatin, silent cold-sensing neurons were unmasked [Fig. 6A(i)]. There was no difference between oxaliplatin-treated mice heterozygous or homozygous for Na_v_1.8-Cre in the cross-sectional areas of cold-responsive cells [Fig. 6A(ii)] or in tdTomato-expression in silent cold-sensing neurons [Fig. 6A(iii)]. Oxaliplatin also evoked cold allodynia in conventional Na_v_1.8 KO mice (Supplementary Fig. 6B). Additionally, imaging of Advillin-Cre conditional Na_v_1.7 KO mice expressing GCaMP3 revealed recruitment of silent cold-sensing neurons by oxaliplatin (Fig. 6B). Thus, pain-related sodium channels Na_v_1.8 and Na_v_1.7 are dispensable for silent cold-sensing neuron activity. Treatment of oxaliplatin-injected animals with TTX blocked activity in essentially all basal and silent cold-sensing neurons, however (Fig. 6C). 4,9-AnhydrousTTX, reported to preferentially inhibit Na_v_1.6, reduced the number of silent cold-sensing neurons by 57%. The effect of Na_v_1.6 blockade on basal cold-sensing neurons was comparable to saline (Fig. 6A and C). Hence, Na_v_1.6 is likely to be the predominant sodium channel isoform in silent cold-sensing neurons. When we directly activated sodium channels with the pharmacological potentiator veratridine in naïve mice, we observed no unmasking of large-sized cells; indeed, the activity of cold-sensing neurons was paradoxically reduced (Fig. 6D). Activation of sodium channels is therefore not sufficient to induce *de novo* cold sensitivity.

**Figure 7:**
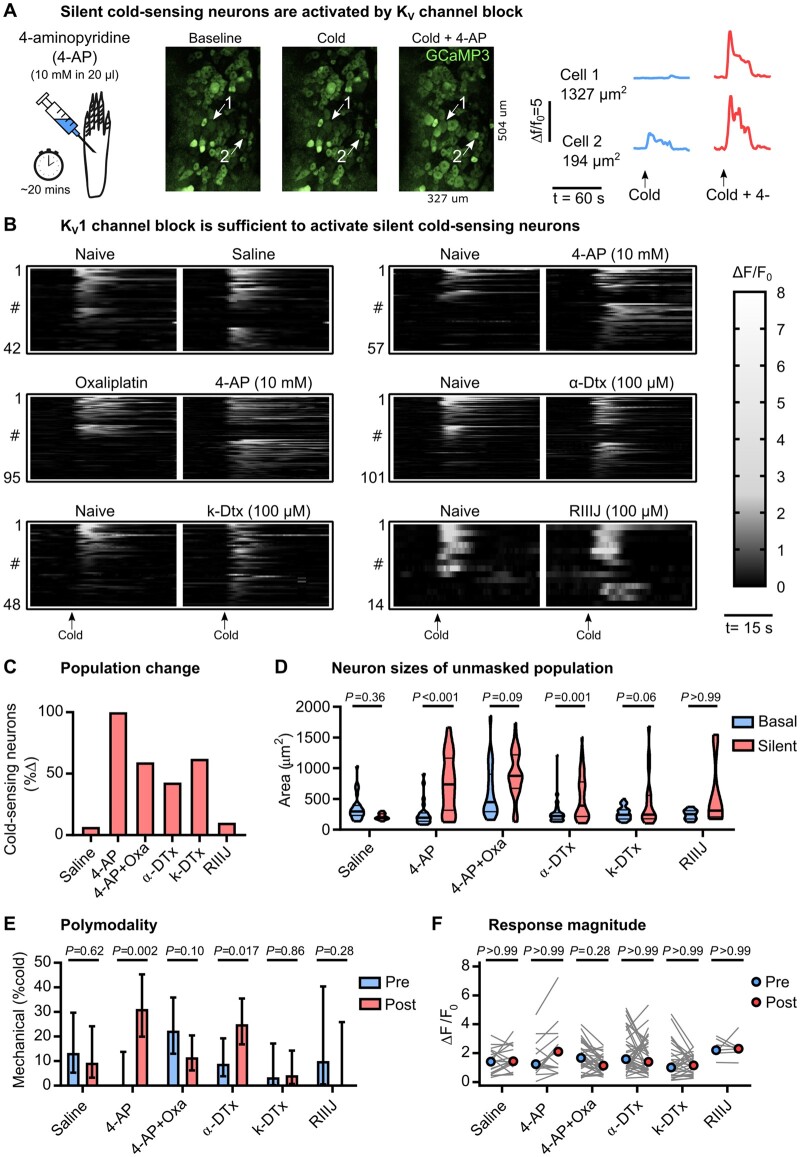
**Blocking K_v_1.1 voltage-gated potassium channels is sufficient to induce *de novo* cold sensitivity in silent cold-sensing neurons.** (**A**) Examples images and traces showing that peripheral blockade of voltage-gated potassium channels induces novel cold-sensitivity in normally cold-insensitive sensory neurons (Cell 1). (**B**) Heat maps showing the effect of intraplantar injection of different potassium channel blockers on the peripheral representation of cold. The bar denotes 15 s. (**C**) Quantification showing the change in the number of cold-sensing neurons after treatment with different potassium channel blockers. (**D**) Violin plots showing the cross-sectional area of basal cold-sensing neurons in the naïve state (blue) and of silent cold-sensing neurons unmasked by potassium channel block (red). Medians were compared using Kruskall-Wallis test followed by Dunn’s multiple comparison test. (**E**) Bar plot of the percentage of polymodal cold-sensing neurons that also respond to noxious mechanical stimuli before (blue) and after (red) treatment with potassium channel blockers. Proportions were compared using a χ^2^ test. Error bars denote 95% confidence intervals. (**F**) No change in the median response magnitude of neurons that responded to cold both before (blue) and after (red) treatment with potassium channel blockers, as determined by Kruskall-Wallis test followed by Dunn’s multiple comparison test. *n* = 42 from three saline-treated mice (one male and two females), *n* = 57 from six 4-AP-treated mice (five males and one female), *n* = 95 from three 4-AP-treated mice pre-injected with oxaliplatin (two males and one female), *n* = 101 from four α-dendrotoxin-treated mice (two males and two females), *n* = 48 from three k-dendrotoxin-treated mice (one male and two females), and *n* = 14 from three RIIIJ-treated mice (three females).

We have previously shown that basally cold-insensitive Na_v_1.8-positive neurons are enriched with *Kcna1* and *Kcna2*, which encode the voltage-gated potassium channels K_v_1.1 and K_v_1.2.^14^ These channels are thought to pass a voltage-dependent hyperpolarizing brake current that opposes depolarization evoked by cooling.^36^ We hypothesized that pharmacological block of the K_v_1 current *in vivo* would therefore unmask silent cold-sensing neurons. We imaged sensory neuron responses to cooling in Pirt-GCaMP3 mice at baseline and 30 min after intraplantar injection of the non-specific voltage-gated potassium channel blocker 4-aminopyridine (4-AP, 10 mM in 20 µl) (Fig. 7A). 4-AP treatment triggered *de novo* sensitivity to cooling in previously cold-insensitive large diameter neurons (Fig. 7A–D). Intriguingly, the effect of 4-AP was reduced by pretreatment with oxaliplatin (Fig. 7B–D).

**Figure 8:**
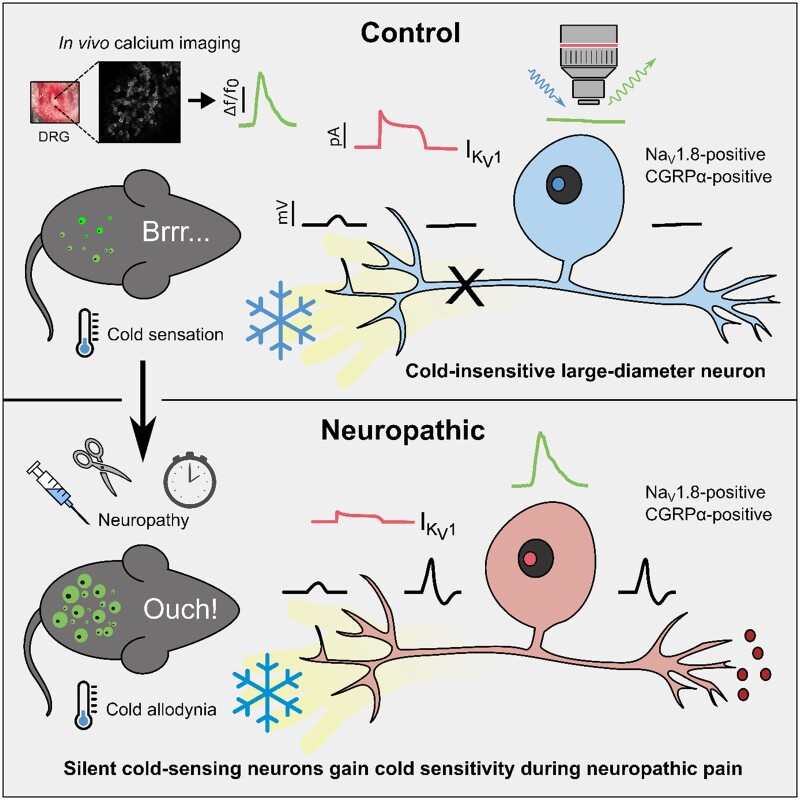
**Proposed model of silent cold-sensing neuron activation during neuropathy to cause cold allodynia.** In healthy mice, only small neurons respond to cold. The large-diameter silent cold-sensing neurons have high K_v_1 activity, thus cold-induced terminal depolarization does not trigger action potential firing and there is no subsequent GCaMP signal at the level of the dorsal root ganglion. In neuropathic animals, we hypothesize that a functional reduction in K_v_1 activity means that silent cold-sensing neurons are now sensitive to cold, increasing nociceptive input to the brain in response to cooling. Thus, both small and large neurons now show GCaMP signals to cold stimuli.

Treatment with the selective K_v_1 antagonist α-dendrotoxin (100 µM) mimicked the effect of 4-AP (Fig. 7B–D), indicating block of K_v_1 channels alone is sufficient to induce *de novo* cold sensitivity in silent cold-sensing neurons. A specific blocker of K_v_1.1, k-dendrotoxin, at 100 µM largely recapitulated the effect of α-dendrotoxin on silent cold-sensing neurons (Fig. 7B–D). The K_v_1.2 blocker conotoxin kM-RIIIJ (100 µM) had only minor effects (Fig. 7B–D). 4-AP and α-dendrotoxin, but not k-dendrotoxin, increased the number of mechano-cold polymodal neurons (Fig. 7E). Interestingly, no potassium channel blocker modified the activity of the basally active population of cold neurons (Fig. 7F). Overall, these data suggest that a functional reduction in K_v_1 channels, primarily mediated through K_v_1.1, could act as a molecular switch to trigger *de novo* cold sensitivity in silent cold-sensing neurons and therefore may also contribute to their unmasking during neuropathic pain (Fig. 8).

## Discussion

### Silent cold-sensing neurons become active during neuropathic pain

Here we have found using *in vivo* imaging that the activation of normally silent, large-diameter silent cold-sensing neurons is a common mechanism of cold allodynia in three models of clinically important neuropathic pain states. The silent cold-sensing neurons identified here typically had functional and molecular characteristics consistent with peptidergic A-fibre nociceptors.^37^ Crucially, we observed no changes in cold thresholds in the basally active population of cold-sensing neurons. Thus, our findings suggest cold allodynia is a form of peripheral sensitization where a subpopulation of nociceptors gains an inappropriate sensitivity to the cold. This contrasts with tactile allodynia, which depends on peripheral drive from low-threshold mechanoreceptors expressing Piezo2.^38–40^

Electrophysiological recordings of both rodent and human nerves exposed to oxaliplatin reveals myelinated A-fibres fire more in the cold.^27^^,^^41^^,^^42^ Blockade of large fibres abolishes cold allodynia in humans with non-freezing cold injury and oxaliplatin neuropathy.^43^^,^^44^ Nerve injury and ciguatoxin likewise increase the fraction of cold-sensitive cutaneous sensory neurons *in vitro.*^20^^,^^24^ There is a gradation—fewer silent cold-sensing neurons are recruited by nerve injury compared to oxaliplatin or ciguatoxin-2, and cold allodynia is correspondingly less prevalent in these patients.^2^^,^^3^ Nonetheless, because silent cold-sensing neurons are unmasked in all three neuropathic pain states tested here, our results do support a common underlying pathophysiology.

In the healthy state, a sparse and modality-specific subpopulation of small-diameter sensory neurons signals cooling.^4^^,^^8^^,^^9^ During neuropathy we find this ‘labelled line’ breaks down, with large neurons responding to both cold and noxious mechanical pinch but rarely to heat, touch and punctate mechanical stimuli. The percentage of silent cold-sensing neurons sensitive to pinch is likely an underestimate due to the differing receptive field areas of these two stimuli. Interestingly, inflammatory mediators also increase the number of polymodal neurons.^4^ Enhanced polymodality is thus a general feature of sensitized pain states.^45^

### Molecular identity of silent cold-sensing neurons

Despite their large size, silent cold-sensing neurons did not express molecular markers for Aβ (*Calb1*) and Aδ (*Ntrk2*) low-threshold mechanoreceptors. This was surprising, given that oxaliplatin preferentially modulates A-fibre activity.^27^^,^^41–43^ However, our findings are consistent with the essential role of *Ntrk2*-positive neurons in tactile but not cold allodynia, indicating the distinct modalities of allodynia are mechanistically different.^46^^,^^47^

The majority of silent cold-sensing neurons expressed the nociceptor marker Na_v_1.8.^29^ Although Na_v_1.8 is not a selective marker of silent cold-sensing neurons, very few of the small diameter, basal cold-sensing cells express this sodium channel.^14^ Thus DTA-mediated ablation of Na_v_1.8-positive neurons in healthy mice had no effect on the moderate cold assays used here to examine allodynia. But after oxaliplatin treatment, deletion of Na_v_1.8-positive neurons including the newly unmasked cold sensors did result in diminished cold allodynia, mechanistically linking silent cold-sensing neurons with cold allodynia. Consistent with this, Na_v_1.8-DTA mice were previously shown to have deficient cold allodynia elicited by ciguatoxin-1, while deletion of HCN2 channels specifically in Na_v_1.8-positive neurons impairs cold allodynia in chronic constriction injury.^24^^,^^48^

We found that most silent cold-sensing neurons express CGRPα, but not Tmem45b, and are therefore likely to be peptidergic nociceptors. Large-diameter, NF200-expressing neurons that are CGRPα-positive, but Trpv1-negative, have been implicated in mechanical nociception, in tune with our finding that a subset of silent cold-sensing neurons responded to pinch.^49^ Indeed, *in vivo* imaging of the trigeminal ganglion has shown that CGRPα-positive neurons are a mixture of small-diameter polymodal nociceptors and large-diameter mechanonociceptors that respond to noxious mechanical stimulation.^50^ Another trigeminal imaging study revealed that, following burn injury of the oral cavity, previously ‘silent’ CGRPα-positive neurons became newly sensitive to cooling.^9^ Tellingly, optogenetic inhibition of CGRPα-positive neurons transiently and reversibly relieves cold allodynia after spared nerve injury.^51^

Taken together, these results identify silent cold-sensing neurons as mainly peptidergic nociceptors that express *Scn10a* and *Calca* molecular markers. Extending our previous observations that Na_v_1.8-positive sensory neurons signal prolonged and extreme cold, a further potential role for these nociceptors in mediating pathological responses to normal cooling is now apparent.^14^

### Ionic mechanisms of *de novo* cold sensitivity

We previously found that voltage-gated potassium channels K_v_1.1 and K_v_1.2 were enriched in basally cold-insensitive, Na_v_1.8-positive neurons.^14^ Silent cold-sensing neurons therefore have high baseline expression of K_v_1 channels which pass a voltage-dependent excitability brake current opposing cold-induced depolarization.^15^^,^^20^ Blocking K_v_1 voltage-gated potassium channels with 4-AP or α-dendrotoxin consequently induced *de novo* cold sensitivity in silent cold-sensing neurons, and this effect was partially recapitulated by inhibiting K_v_1.1 but not K_v_1.2. Although oxaliplatin and P-CTX-2 directly activate voltage-gated sodium channels, potentiating sodium channels does not drive *de novo* cold-sensitivity, indicating that ectopic cold activation is not a consequence of a general increase in excitability but specifically linked to inhibiting K_v_1 channels.^26^^,^^27^ Indeed, K_v_1 channels are known to control action potential firing potently in response to sensory stimuli in both cold- and mechanically sensitive nerve terminals.^15^^,^^52^

Does neuropathic pain lead to functional downregulation of K_v_1 channels? 4-AP induces *de novo* cold sensitivity in sensory neurons from control but not nerve injured mice.^20^ 4-AP evoked behavioural cold hypersensitivity is also suppressed in injured animals, indicating that nerve injury-induced cold allodynia operates via the same pathway as 4-AP to drive *de novo* cold sensitivity.^20^ Corroborating this, we found that 4-AP unmasked fewer silent cold-sensing neurons in mice pretreated with oxaliplatin. The mechanism of K_v_1 channel of downregulation is unclear and likely to be specific to each disease state. Quantitative PCR of samples from oxaliplatin-treated mice reveal that there is a decrease in K_v_1.1 RNA, supporting transcriptional changes.^19^ Numerous reports have also found a decrease in both K_v_1.1 and K_v_1.2 expression following nerve injury.^53–59^ On the other hand, *in vitro* studies support a direct antagonist effect of both oxaliplatin and ciguatoxin on voltage-gated potassium channels.^42^^,^^60^ However, it is important to note that a causal link between peripheral neuropathy and K_v_1 channel activity was not investigated or explicitly demonstrated in our study.

## Conclusions

Overall, we show that cold allodynia results from a set of normally silent cold-sensing neurons gaining *de novo* cold sensitivity in neuropathic pain. Cold allodynia is therefore a form of peripheral sensitization. Silent cold-sensing neurons were identified as putative A-fibre peptidergic nociceptors based on their large diameter, response to noxious mechanical stimulation and expression of molecular markers Na_v_1.8 and CGRPα. Block of K_v_1 channels is sufficient to induce *de novo* cold sensitivity, pointing to the downregulation of these channels during disease as a possible trigger of cold allodynia. By defining cells and molecules involved in cold allodynia, our findings will inform the development of better targeted therapeutics for neuropathic pain. The *in vivo* imaging data collected here provide a unique insight into the mechanisms underpinning cold allodynia, for the first time identifying silent cold-sensing neurons as critical drivers of cold-evoked neuropathic pain.

## Supplementary Material

awab086_Supplementary_Data

## Abbreviations

PNL: partial sciatic nerve ligation
