# Supplementary material for: Silent cold-sensing neurons contribute to cold allodynia in neuropathic pain
Source: Brain. 2021 Mar 9;144(6):1711–26. doi: 10.1093/brain/awab086 (PMC8320254; doi:10.1093/brain/awab086)
Supplement: awab086_Supplementary_Data [file awab086_supplementary_data.pdf]

# 1 Supplementary Methods

## 1.1 Behavioural Testing

All animal procedures were approved by UCL ethical review committees and were discussed with Home Office inspectors to conform to UK Home Office regulations under Project licence P413329A2. The investigator was blinded to treatment and/or genotype. Animals were acclimatized to handling and every effort was made to minimize stress during the testing. Both male and female animals were used. All experiments were performed at a room temperature of between 18 and 21°C.

### 1.1.1 Von Frey:

Punctate mechanical sensitivity was measured using the up-down method to obtain a 50% withdrawal threshold (Chaplan *et al.*, 1994). Mice were habituated for one hour in darkened enclosures with a wire mesh floor. A 0.4 g von Frey filament was applied to the plantar surface of the paw for 3 s. A positive response resulted in application of a filament of lesser strength on the following trial, and no response in application of a stronger filament. To calculate the 50% withdrawal threshold, five responses surrounding the 50% threshold were obtained after the first change in response. The pattern of responses was used to calculate the 50% threshold = $(10[\chi+\kappa\delta])/10{,}000$), where $\chi$ is the log of the final von Frey filament used, $\kappa$ = tabular value for the pattern of responses and $\delta$ the mean difference between filaments used in log units. The log of the 50% threshold was used to calculate summary and test statistics, in accordance with Weber's Law.

### 1.1.2 Hot Plate:

The Hot Plate test measures supraspinal nociceptive behaviours in response to extreme heat (Woolfe and MacDonald, 1944). Mice were placed on the Hot Plate apparatus held at 50°C or 55°C. The tested ended when the animal showed a withdrawal behavior or licked its hindpaw. Cut-off time was 60 s for 50°C and 30 s for 55°C.

### 1.1.3 Cold Plate:

Mice were placed on the Cold Plate apparatus (Allchorne *et al.*, 2005), surrounded by a Perspex glass enclosure. The Cold Plate was maintained at 5°C for 5 minutes while the animal was free to move around on the plate and the number of nociceptive and nocifensive behaviours (shaking, lifting, licking, guarding, biting) displayed by each paw were counted by the observer (Deuis *et al.*, 2013).

### 1.1.4 Unilateral Cold Plate:

Mice were acclimatized to handling and scruffing in the days leading up to the baseline experiment. During the test, the animals was restrained by lightly scruffing the nape of the neck, ensuring the animal was calm and not showing non-specific movements of the hindlimbs. The ipsilateral hind-paw was placed directly onto the plate maintained at 10°C, with the

contralateral hind-paw placed on an adjoining thermoneutral surface. The animal was held vertically such that its forepaws did not contact any surface. The time until ipsilateral hindpaw withdrawal was then measured. This was repeated two to three times and a trial averaged latency to withdrawal was obtained.

### 1.1.5 Acetone Test:

The acetone evaporation test measures the nociceptive behaviours triggered by evaporative cooling of the hindpaw (Yoon *et al.*, 1994). Mice were habituated for 1 hour in enclosures with a wire mesh floor. Using a home-made syringe, a 50 µl drop of acetone was applied to the ventral side of the ipsilateral hindpaw. The cumulative time where the ipsilateral hindpaw was engaged in nociceptive and nocifensive behaviours (lifting, shaking, licking, guarding, biting) over the ensuing 60 s was then counted. An average of two to three trials was obtained, with at least 10 minutes between trials

## 1.2 *In vitro* electrophysiology

### 1.2.1 DRG neuronal culture

Adult mice were killed by inhalation of a rising $CO_2$ concentration followed by cervical dislocation to confirm death. Dorsal root ganglia (DRG) were dissected from the entire length of the spinal column and then digested in a pre-equilibrated enzyme mix for 45 minutes (37 ˚C, 5% $CO_2$). The enzyme mix consisted of Hanks' balanced salt solution containing collagenase (type XI; 5 mg/ml), dispase (10 mg/ml), HEPES (5 mM) and glucose (10 mM). DRGs were then gently centrifuged for 5 minutes at 300 revolutions per minute, the supernatant was discarded and replaced with warmed Dulbecco's modified Eagle's medium (DMEM), supplemented with L-glutamine (1%), glucose (4.5 g/litre), sodium pyruvate (110 mg/litre) and 10% fetal bovine serum (FBS). Next, DRGs were mechanically triturated with three fire-polished glass Pasteur pipettes of gradually decreasing inner diameter. Dissociated cells were then centrifuged again at 300 revolutions per minute, the supernatant was discarded and cells were re-suspended in the required volume of DMEM supplemented with FBS and nerve growth factor (50 ng/ml). Finally, cells were plated onto 12 mm glass coverslips coated with poly-L-lysine (1 mg/ml) and laminin (1 mg/ml). Cells were incubated at 37 ˚C in 5% $CO_2$ and recordings were performed at room temperature (18-21 ˚C) between 24 – 72 hours after dissociation.

### 1.2.2 Whole cell patch-clamp

Functional deletion of $Na_V1.8$ was assessed by pharmacological isolation of TTX-resistant currents. Patch pipettes (tip resistance of 3-5 MΩ) were filled with intracellular solution containing: 140 mM CsF, 1 mM EGTA, 5 mM NaCl, 10 mM HEPES. Neurons were perfused with extracellular solution containing in: 70 mM NaCl, 70 mM Choline-Cl, 3 mM KCl, 1 mM $MgCl_2$, 20 mM TEA-Cl, 0.1 mM $CdCl_2$, and 10 mM Glucose. 5nM TTX was included in the extracellular solution to isolate TTX-resistant currents. Whole-cell recordings were obtained using an Axopatch 200B amplifier, filtered at 10 kHz and digitized at 50 kHz via a Digidata

1322A (Axon Instruments). tdTomato-expressing neurons from heterozygous and homozygous $Na_V1.8$-Cre mice were voltage-clamped at -70 mV. Series resistance compensation was at least 60%. To measure the voltage-dependence of sodium channel activation, the holding command was dropped to -120 mV to de-inactivate all sodium channels and then a step-protocol from -80 to 20 mV was applied, in increments of 5 mV, to activate sodium channels.

# 2 Supplementary Figures

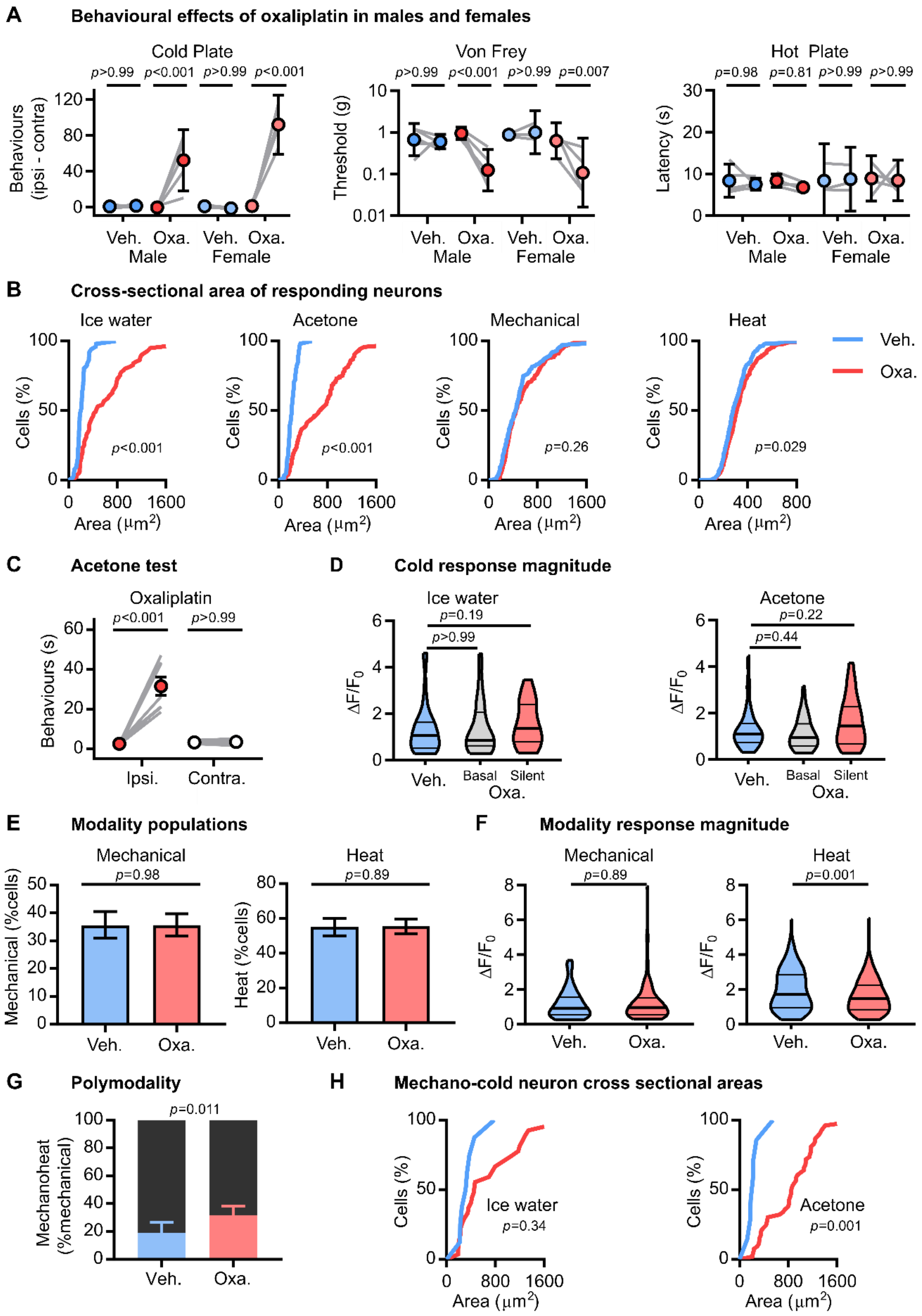

**Supplementary Fig. 1. Behavioural and functional effects of oxaliplatin treatment.**

(A) Comparison of the behavioural effects of oxaliplatin on different sensory modalities (cold, mechanical and heat) in male and female mice. For vehicle, n=5 males and n=3 females. For oxaliplatin, n=5 males and n=4 females. Means were before and after treatment were compared by 2-way ANOVA followed by post-hoc Sidak's test. Error bars denote 95% confidence interval.

(B) Cumulative probability plots of cross-sectional areas for cells responding to each stimulus modality, compared using Kolmogorov-Smirnov test.

(C) Effect of oxaliplatin on acetone-evoked pain behaviour. Means before and after treatment were compared using repeated measures 2-way ANOVA with post-hoc Sidak's test. n=7.

(D) Violin plots showing the peak responses evoked by cold stimuli in the vehicle group and separately in the basal and silent cold-sensing neurons from the oxaliplatin group. Ice water: n=51 for vehicle, n=40 for basal, and n=41 for silent. Acetone: n=58 for vehicle, n=57 for basal, and n=88 for silent. Medians were compared by Kruskall-Wallis test followed by Dunn's multiple comparison's test.

(E) Bar plots showing the proportion of all responding neurons responding to heat or mechanical stimuli, compared using $\chi^2$ test. Error bars denote 95% confidence interval.

(F) Violin plots showing peak response evoked by each stimulus modality, compared using Mann-Whitney test.

(G) Proportion of mechanically-sensitive neurons also responding to noxious heat, compared using $\chi^2$ test. Error bars denote 95% confidence interval.

(H) Cumulative probability plots showing mechano-cold neurons tend to have larger cross-sectional areas in the oxaliplatin group, compared using the Kolmogorov-Smirnov test. Ice water: $n_{veh}$=8, $n_{oxa}$=27. Acetone: $n_{veh}$=7, $n_{oxa}$=53.

Ice-water: $n_{veh}$=51, $n_{oxa}$=81. Acetone: $n_{veh}$=58, $n_{oxa}$=145. Mechanical: $n_{veh}$=136, $n_{oxa}$=193. Heat: $n_{veh}$=211, $n_{oxa}$=301.

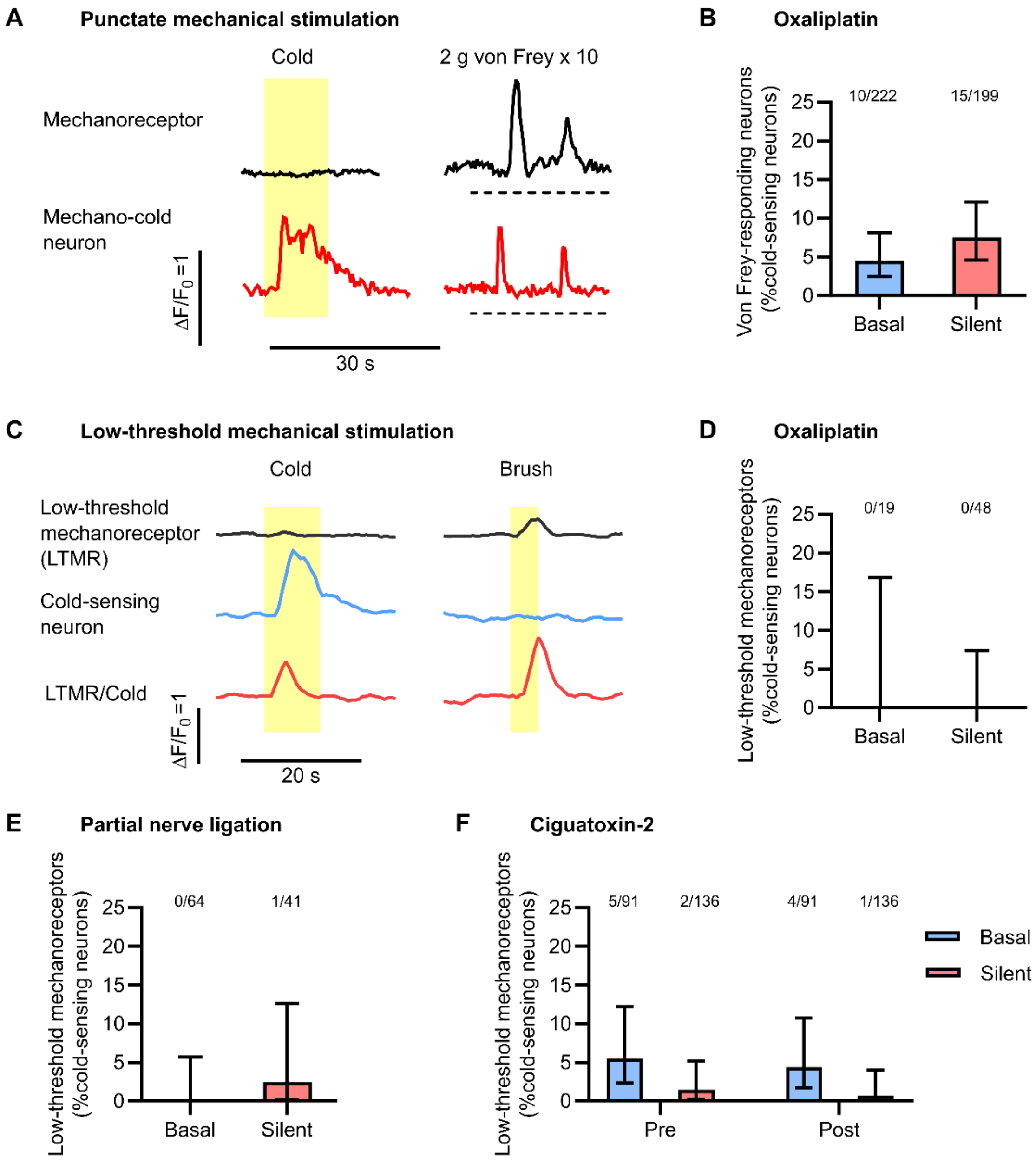
A
Punctate mechanical stimulation
Cold
2 g von Frey x 10
Mechanoreceptor
Mechano-cold
neuron
ΔF/F0 =1
30 s
B
Oxaliplatin
Von Frey-responding neurons
(%cold-sensing neurons)
10/222
15/199
25
20
15
10
5
0
Basal
Silent
C
Low-threshold mechanical stimulation
Cold
Brush
Low-threshold
mechanoreceptor
(LTMR)
Cold-sensing
neuron
LTMR/Cold
ΔF/F0 =1
20 s
D
Oxaliplatin
Low-threshold mechanoreceptors
(%cold-sensing neurons)
0/19
0/48
Basal
Silent
E
Partial nerve ligation
Low-threshold mechanoreceptors
(%cold-sensing neurons)
0/64
1/41
Basal
Silent
F
Ciguatoxin-2
Low-threshold mechanoreceptors
(%cold-sensing neurons)
5/91
2/136
4/91
1/136
Basal
Silent
Pre
Post

**Supplementary Fig. 2. Silent cold-sensing neurons are not low-threshold mechanoreceptors.**

(A) Representative traces of DRG neuron calcium signals in an oxaliplatin-treated mouse in response to cold and 2g Von Frey stimuli.

(B) Bar plot showing the percentage of basal and silent cold-sensing neurons in oxaliplatin-treated mice that show a response to repeated 2 g Von Frey stimulation. n=421 cold-sensing neurons from 19 oxaliplatin-treated animals (10♂ & 9♀).

(C) Example traces of DRG neuron calcium signals in a naive mouse in response to cold and brush stimuli. Example traces of a low-threshold mechanoreceptor (top) and a cold-sensing neurons (middle) are shown. A rare neuron responding to both brushing and cooling is also shown (bottom).

(D) Bar plot showing the percentage of basal and silent cold-sensing neurons in oxaliplatin-treated mice that show any response to a wide range of low-threshold mechanical stimuli (cotton swap/brush was applied to glabrous skin and to hairy skin with and against grain). n=67 cold-sensing neurons from 3 oxaliplatin-treated animals (2♂ & 1♀).

(E) Bar plot showing the percentage of basal and silent cold-sensing neurons in mice with partial nerve ligation that show a response to brushing of the paw. n=105 cold-sensing neurons from 6 PNL-operated animals (3♂ & 3♀).

(F) Bar plot showing the percentage of basal and silent cold-sensing neurons in P-CTX-2-treated mice that show a response to brush either before or after treatment. n=227 cold-sensing neurons from 10 P-CTX-2-injected mice (4♂ & 6♀).

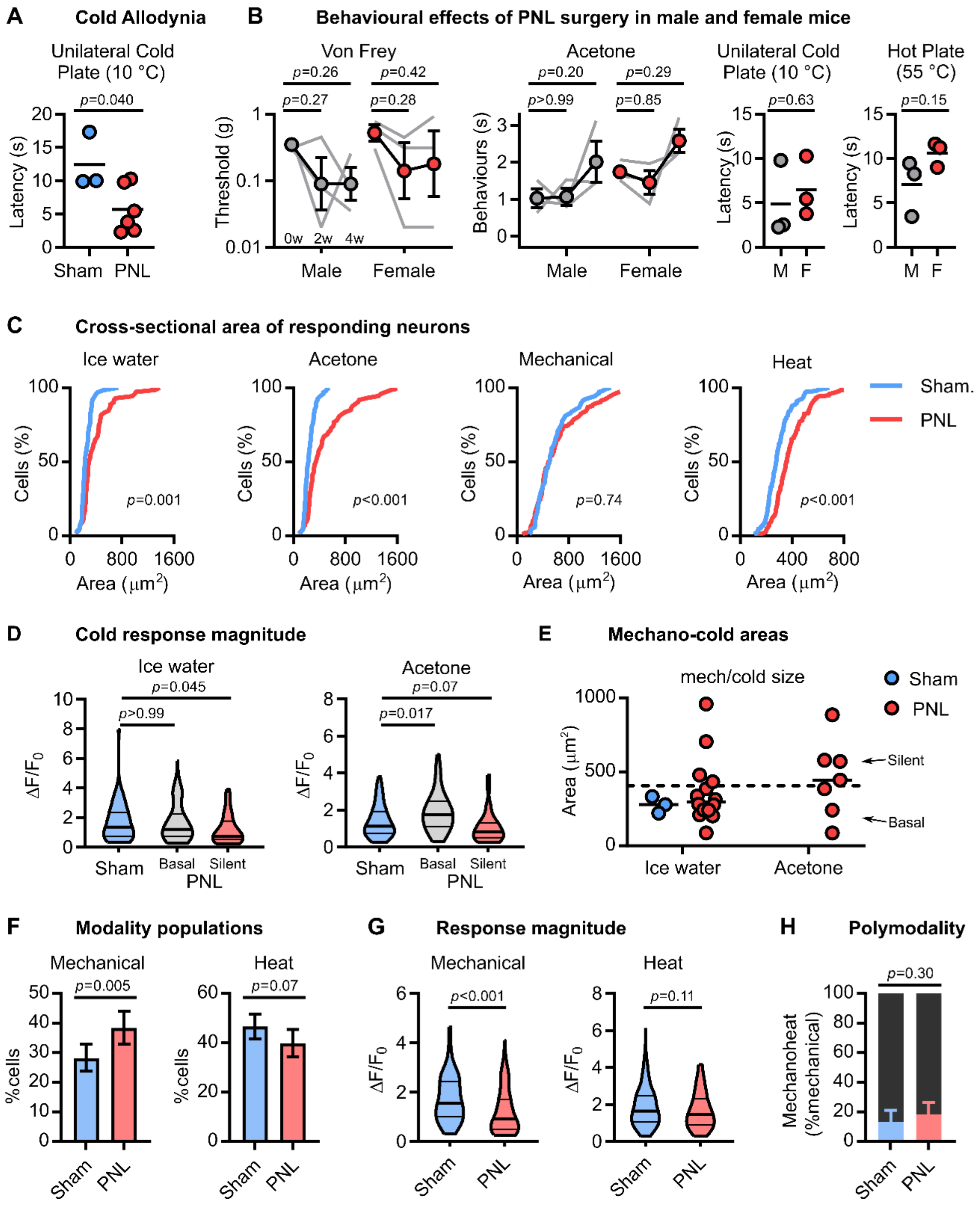

A Cold Allodynia
B Behavioural effects of PNL surgery in male and female mice
Unilateral Cold Plate (10 °C)
p=0.040
Latency (s)
Sham PNL
Von Frey
p=0.26 p=0.42
p=0.27 p=0.28
Threshold (g)
0w 2w 4w
Male Female
Acetone
p=0.20 p=0.29
p>0.99 p=0.85
Behaviours (s)
Unilateral Cold Plate (10 °C)
p=0.63
Hot Plate (55 °C)
p=0.15
M F
C Cross-sectional area of responding neurons
Ice water
Acetone
Mechanical
Heat
Cells (%)
Area (μm²)
p=0.001
p<0.001
p=0.74
Sham.
PNL
D Cold response magnitude
Ice water
p=0.045
p>0.99
Acetone
p=0.07
p=0.017
ΔF/F₀
Sham Basal Silent
PNL
E Mechano-cold areas
mech/cold size
Area (μm²)
Silent
Basal
Ice water Acetone
F Modality populations
Mechanical
p=0.005
Heat
p=0.07
%cells
G Response magnitude
p<0.001
p=0.11
H Polymodality
p=0.30
Mechanoheat (%mechanical)

**Supplementary Fig. 3. Behavioural and functional effects of partial nerve ligation.**

(A) Latency to withdrawal on Unilateral Cold Plate four weeks after sham or PNL surgery.. Means were compared using two-tailed unpaired *t* test. n=3 (1♂ & 2♀) for sham and n=6 (3♂ & 3♀) for PNL.

(B) Comparison of the behavioural effects of partial nerve ligation (PNL) on different sensory modalities (cold, mechanical and heat) in male and female mice. For PNL, n=3 males and n=3 females. Means were before and after treatment were compared by 2-way ANOVA followed by post-hoc Sidak's test, or using a two-tailed unpaired *t* test. Error bars denote standard error of the mean.

(C) Cumulative probability plots of cross-sectional areas for cells responding to each stimulus modality, compared using Kolmogorov-Smirnov test.

(D) Violin plots showing the peak responses evoked by cold stimuli in the sham group and separately in the basal and silent cold-sensing neurons for the PNL group. Ice water: n=64 for sham, n=46 for basal, and n=25 for silent. Acetone: n=95 for sham, n=42 for basal, and n=31 for silent. Medians were compared by Kruskall-Wallis test followed by Dunn's multiple comparison's test.

(E) Scatter plots showing mechano-cold neurons have both small and large cross-sectional areas in the PNL group. Mech./ice water: $n_{sham}$=3, $n_{PNL}$=14. Mech./acetone: $n_{sham}$=0, $n_{PNL}$=7.

(F) Bar plots showing the proportion of all responding neurons responding to heat or mechanical stimuli, compared using $\chi^2$ test. Error bars denote 95% confidence interval.

(G) Violin plots showing peak response evoked by each stimulus modality, compared using Mann-Whitney test.

(H) Proportion of mechanically-sensitive neurons also responding to noxious heat, compared using $\chi^2$ test.

Ice-water: $n_{sham}$=64, $n_{PNL}$=71. Acetone: $n_{sham}$=95, $n_{PNL}$=73. Mechanical: $n_{sham}$=105, $n_{PNL}$=114. Heat: $n_{sham}$=174, $n_{PNL}$=118.

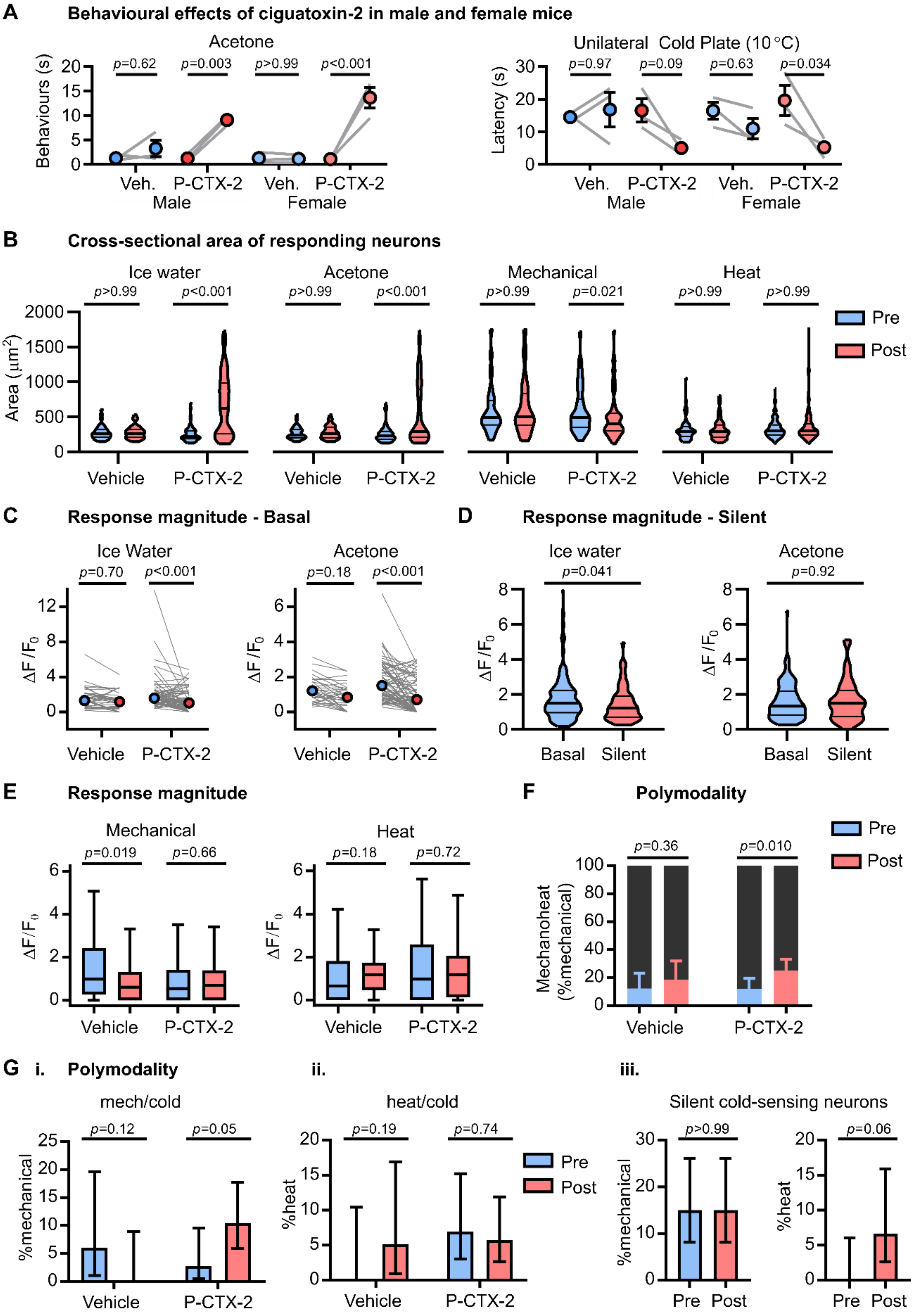
A
Behavioural effects of ciguatoxin-2 in male and female mice
Acetone
Behaviours (s)
p=0.62
p=0.003
p>0.99
p<0.001
Veh.
P-CTX-2
Male
Female
Unilateral Cold Plate (10 °C)
Latency (s)
p=0.97
p=0.09
p=0.63
p=0.034
B
Cross-sectional area of responding neurons
Ice water
Acetone
Mechanical
Heat
Area (μm²)
p>0.99
p<0.001
p=0.021
Vehicle
P-CTX-2
Pre
Post
C
Response magnitude - Basal
Ice Water
p=0.70
p<0.001
p=0.18
ΔF/F0
D
Response magnitude - Silent
Ice water
p=0.041
p=0.92
Basal
Silent
E
Response magnitude
Mechanical
p=0.019
p=0.66
Heat
p=0.18
p=0.72
F
Polymodality
p=0.36
p=0.010
Mechanoheat (%mechanical)
G
i.
Polymodality
mech/cold
p=0.12
p=0.05
%mechanical
ii.
heat/cold
p=0.19
p=0.74
%heat
iii.
Silent cold-sensing neurons
p>0.99
p=0.06

**Supplementary Fig. 4. Behavioural and functional effects of ciguatoxin-2.**

(A) Behavioural effects of ciguatoxin-2 on cold sensitivity in male ($n_{veh}$=3; $n_{P\text{-}CTX\text{-}2}$=3) and female mice ($n_{veh}$=3; $n_{P\text{-}CTX\text{-}2}$=3). Error bars denote standard error of the mean.

(B) Violin plots of cross-sectional areas for cells responding to each stimulus modality, compared using Kruskall-Wallis test followed by Dunn's multiple comparisons test.

(C) Line plots showing the median response magnitude of basally cold-sensitive neurons before and after treatment, compared using Kruskall-Wallis test followed by Dunn's multiple comparisons test.

(D) Violin plots showing the response magnitude of all silent cold-sensing neurons unmasked by P-CTX-2 (n=127 for ice-water, and n=60 for acetone) compared to all basally-active neurons recorded from naïve mice (n=105 for both). Medians were compared using Mann-Whitney test.

(E) Box plots showing the median response magnitude of all mechanical and heat-responsive neurons before and after treatment.

(F) Proportion of mechanically-sensitive neurons also responding to noxious heat, before and after treatment, compared using $\chi^2$ test. Error bars denote 95% confidence intervals.

(G) Quantification of the proportion of neurons responding acetone that were also sensitive to either mechanical (i.) or heat (ii.) before and after treatment. (iii.) Comparison of the proportion of silent cold-sensing neurons activated by acetone that were responsive to other modalities before and after the induction of cold-sensitivity by P-CTX-2. n=60. The proportion of polymodal neurons was compared using $\chi^2$ test, and error bars denote 95% confidence intervals.

Ice-water: vehicle: $n_{pre}$=36, $n_{post}$=43; P-CTX-2: $n_{pre}$=69, $n_{post}$=174. Acetone: vehicle: $n_{pre}$=33, $n_{post}$=39; P-CTX-2: $n_{pre}$=72, $n_{post}$=105. Mechanical: vehicle: $n_{pre}$=57, $n_{post}$=48; P-CTX-2: $n_{pre}$=115, $n_{post}$=131. Heat: vehicle: $n_{pre}$=59, $n_{post}$=77; P-CTX-2: $n_{pre}$=211, $n_{post}$=241.

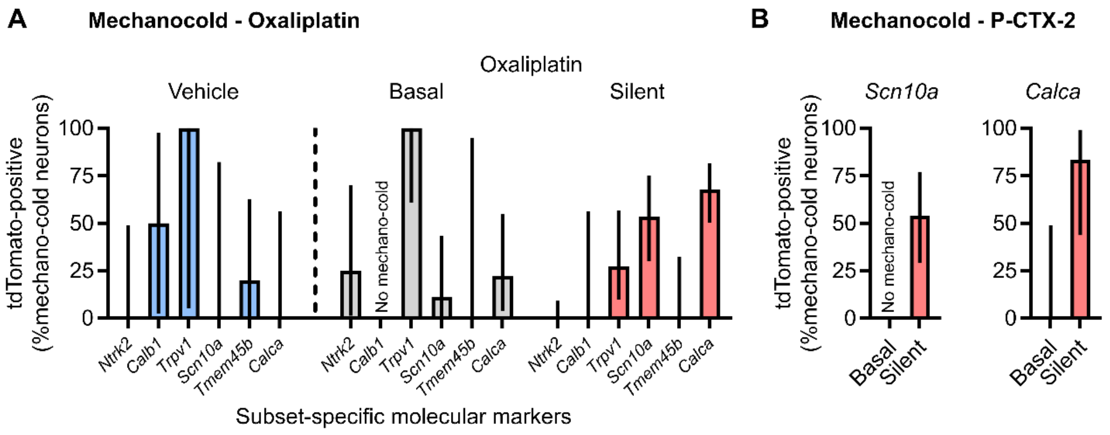


**Supplementary Fig. 5. Molecular characterization of mechanically-sensitive silent cold-sensing neurons.**

(A) Bar plot showing overlap of reporter expression for each marker with polymodal mechano-cold neurons from oxaliplatin-treated and vehicle-treated mice.

TrkB-CreERT2 (*Ntrk2*): $n_{veh}$=4 from 2 mice (1♂ & 1♀)), $n_{oxa}$=42 from 3 mice (2♂ & 1♀). Calb1-Cre (*Calb1*): $n_{veh}$=2 from 1 mouse (1♂), $n_{oxa}$=3 from 2 mice (2♀). Trpv1-Cre (*Trpv1*): $n_{veh}$=1 from 1 mouse (1♂), $n_{oxa}$=17 from 2 mice (1♂ & 1♀). $Na_V$1.8-Cre (*Scn10a*): $n_{veh}$=2 from 4 mice (2♂ & 2♀), $n_{oxa}$=24 from 6 mice (4♂ & 2♀). Tmem45b-Cre (*Tmem45b*): $n_{veh}$=5 from 1 mouse (1♂), $n_{oxa}$=9 from 3 mice (2♂ & 1♀). CGRPα-CreERT2 (*Calca*): $n_{veh}$=3 from 3 mice (1♂ & 2♀), $n_{oxa}$=40 from 2 mice (1♂ & 1♀).

(B) Bar plot showing overlap of *Scn10a* and *Calca* expression with basal and silent mechano-cold neurons from P-CTX-2-treated animals. $Na_V$1.8-Cre (*Scn10a*): n=13 4 mice (1♂ & 3♀). CGRPα-CreERT2 (*Calca*): n=10 from 2 mice (1♂ & 1♀).

Note that too few polymodal mechano-cold neurons were observed in the partial nerve ligation marker dataset for reliable analysis of *Scn10a* and *Calca* expression.

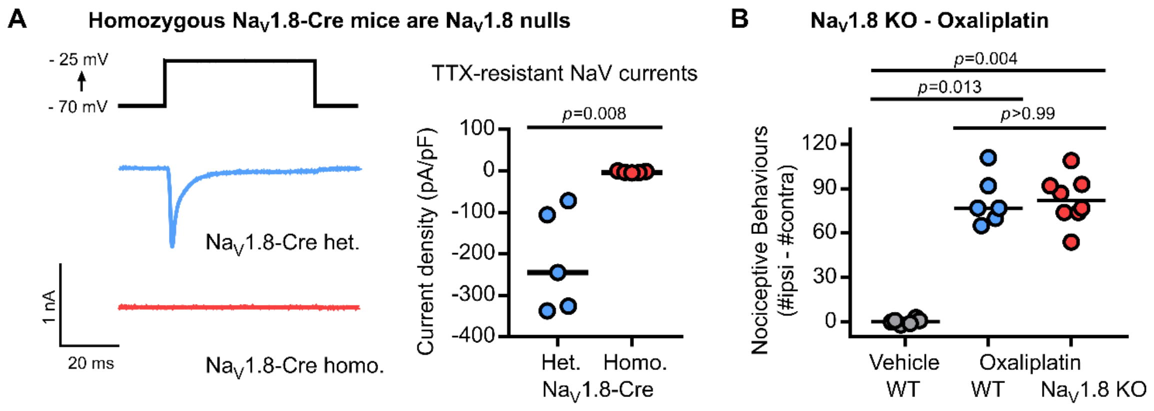


**Supplementary Fig. 6. Mice lacking Na$_V$1.8 develop oxaliplatin-induced cold allodynia.**

(A) Example traces and scatter plot of TTX-resistant sodium currents recorded from medium-sized DRG neurons cultured from heterozygous Na$_V$1.8-Cre mice (blue). No TTX-resistant currents were observed in DRGs from homozygous Na$_V$1.8-Cre mice (red). Median (line) current density was compared using the Mann-Whitney test. n=5 cells from 1 heterozygous mouse, and n=5 cells from 1 homozygous mouse.

(B) Scatter plot of the effect of oxaliplatin on pain behaviours evoked by 5°C cold plate in WT and conventional Na$_V$1.8 KO mice. Medians were compared using Kruskall Wallis test followed by Dunn's multiple comparisons test. n=6 for WT treated with vehicle (3♂ & 3♀). n=6 for WT treated with oxaliplatin (2♂ & 4♀). and n=8 for Na$_V$1.8 KO treated with oxaliplatin (4♂ & 4♀).

# 3 Supplementary Tables

## 3.1 Supplementary Table 1

| MOUSE LINE | IDENTIFIER | CITATION | STOCK |
|---|---|---|---|
| Pirt-GcaMP3 | $Pirt^{tm2Xzd}$ | (Kim *et al.*, 2014) | N/A |
| $Na_V1.7$ flox | $Scn9a^{tm1.1Jnw}$ | (Nassar *et al.*, 2004) | N/A |
| Advillin Cre | B6.129P2-*Avil*$^{tm2(cre)Fawa}$/J | (Zhou *et al.*, 2010) | 032536 |
| Rosa-flox-stop tdTomato (Ai9) | B6;129S6-*Gt(ROSA)26Sor*$^{tm9(CAG-tdTomato)Hze}$/J | (Madisen *et al.*, 2010) | 007905 |
| Calb1-Cre | B6;129S-*Calb1*$^{tm2.1(cre)Hze}$/J | (Nigro *et al.*, 2018) | 028532 |
| TrkB-CreERT2 | B6.129S6(Cg)-*Ntrk2*$^{tm3.1(cre/ERT2)Ddg}$/J | (Rutlin *et al.*, 2014) | 027214 |
| Trpv1-Cre | B6.129-*Trpv1*$^{tm1(cre)Bbm}$/J | (Cavanaugh *et al.*, 2011) | 017769 |
| $Na_V1.8$-Cre | $Scn10a^{tm2(cre)Jnw}$ | (Nassar *et al.*, 2004) | N/A |
| Tmem45b-Cre | $TMEM45b^{CRE}$ | (Cox, J., in preparation) | N/A |
| CGRPα-CreERT2 | $Calca^{tm1.1(cre/ERT2)Ptch}$ | (Song *et al.*, 2012) | N/A |
| $Na_V1.8$ KO | $Scn10a^{tm1Jnw}$ | (Akopian *et al.*, 1999) | N/A |
| Rosa-flox-stop DTA | *Gt(ROSA)26Sor*$^{tm1(DTA)Jpmb}$/J | (Ivanova *et al.*, 2005) | 006331 |

## 3.2 Supplementary Table 2

| FIGURES | DATASET | GENOTYPE | GROUP | M | F | TOTAL |
|---|---|---|---|---|---|---|
| Figure 1 & Supplementary Figure 1 | Oxaliplatin - imaging and behaviour | Pirt-GCaMP3 | Vehicle | 5 | 3 | 8 |
| | | | Oxaliplatin | 5 | 4 | 9 |
| Supplementary Figure 1 | Oxaliplatin - acetone behaviour | Pirt-GCaMP5 | Oxaliplatin | 5 | 2 | 7 |
| Figure 2 & Supplementary Figure 3 | PNL - imaging and behaviour | Pirt-GCaMP6 | Sham | 1 | 2 | 3 |
| | | | PNL | 3 | 3 | 6 |
| Figure 3 & Supplementary Figure 4 | Ciguatoxin - behaviour | Pirt-GCaMP8 | Vehicle | 3 | 3 | 6 |
| | | | P-CTX-2 | 3 | 3 | 6 |
| Figure 3 & Supplementary Figure 4 | Ciguatoxin - imaging | Pirt-GCaMP10 | Vehicle | 2 | 1 | 3 |
| | | | P-CTX-2 | 4 | 6 | 10 |
| Figure 4 & Supplementary Figure 5 | Oxaliplatin - Ntrk2 | TrkB-Cre CreERT2 tdTomato Pirt-GCaMP3 | Vehicle | 1 | 1 | 2 |
| | | | Oxaliplatin | 2 | 1 | 3 |
| Figure 4 & Supplementary Figure 5 | Oxaliplatin - Calb1 | Calb1-Cre tdTomato Pirt-GCaMP3 | Vehicle | 1 | 0 | 1 |
| | | | Oxaliplatin | 0 | 2 | 2 |
| Figure 4 & Supplementary Figure 5 | Oxaliplatin - Trpv1 | Trpv1-Cre tdTomato Pirt-GCaMP3 | Vehicle | 1 | 0 | 1 |
| | | | Oxaliplatin | 1 | 1 | 2 |
| Figure 4 & Supplementary Figure 5 | Oxaliplatin - Scn10a | Nav1.8-Cre tdTomato Pirt-GCaMP3 | Vehicle | 2 | 2 | 4 |
| | | | Oxaliplatin | 4 | 2 | 6 |
| Figure 4 & Supplementary Figure 5 | Oxaliplatin - Tmem45b | Tmem45b-Cre tdTomato Pirt-GCaMP3 | Vehicle | 1 | 0 | 1 |
| | | | Oxaliplatin | 2 | 1 | 3 |
| Figure 4 & Supplementary Figure 5 | Oxaliplatin - Calca | CGRPa-CreERT2 tdTomato Pirt-GCaMP3 | Vehicle | 1 | 2 | 3 |
| | | | Oxaliplatin | 1 | 1 | 2 |
| Figure 4 | PNL - Scn10a | Nav1.8-Cre tdTomato Pirt-GCaMP3 | PNL | 1 | 1 | 2 |
| Figure 4 & Supplementary Figure 5 | Ciguatoxin - Scn10a | Nav1.8-Cre tdTomato Pirt-GCaMP3 | P-CTX-2 | 1 | 3 | 4 |
| Figure 4 | PNL - Calca | CGRPa-CreERT2 tdTomato Pirt-GCaMP3 | PNL | 1 | 0 | 1 |
| Figure 4 & Supplementary Figure 5 | Ciguatoxin - Calca | CGRPa-CreERT2 tdTomato Pirt-GCaMP3 | P-CTX-2 | 1 | 1 | 2 |
| Figure 5 | Ablation of Nav1.8 neurons - imaging | Nav1.8-Cre tdTomato Pirt-GCaMP2 | Oxaliplatin | 4 | 2 | 6 |
| | | Nav1.8-Cre tdTomato DTA Pirt-GCaMP3 | | 1 | 1 | 2 |
| | | Nav1.8-Cre tdTomato Pirt-GCaMP2 | Vehicle | 2 | 2 | 4 |
| Figure 5 | Ablation of Nav1.8 neurons - behaviour | Nav1.8-Cre WT | Oxaliplatin | | | 10 |
| | | Nav1.8-Cre DTA | | | | 8 |
| Figure 6 | Knockout of Nav1.8 - imaging | Nav1.8-Cre het tdTomato Pirt-GCaMP3 | Oxaliplatin | 4 | 2 | 6 |
| | | Nav1.8-Cre homo tdTomato Pirt-GCaMP3 | | 1 | 2 | 3 |

| FIGURES | DATASET | GENOTYPE | GROUP | M | F | TOTAL |
|---|---|---|---|---|---|---|
| Figure 6 | Knockout of Nav1.7 - imaging | Nav1.7 WT | Oxaliplatin | 1 | 4 | 5 |
| | | Advillin-Cre Nav1.7 KO | | 0 | 2 | 2 |
| Figure 6 | Sodium channel blockers - imaging | Pirt-GCaMP3 | Oxaliplatin + Saline | 1 | 2 | 3 |
| | | | Oxaliplatin + TTX | 3 | 1 | 4 |
| | | | Oxaliplatin + anhyTTX | 0 | 2 | 2 |
| Figure 6 | Sodium channel activation - imaging | Pirt-GCaMP3 | Veratridine | 3 | 0 | 3 |
| Figure 7 | Potassium channel blockers - imaging | Pirt-GCaMP3 | Saline | 1 | 2 | 3 |
| | | | 4-AP | 5 | 1 | 6 |
| | | | 4-AP + Oxa | 2 | 1 | 3 |
| | | | aDTx | 2 | 2 | 4 |
| | | | kDTx | 1 | 2 | 3 |
| | | | RIIIJ | 0 | 3 | 3 |
| Supplementary Figure 2 | Von Frey testing - imaging | Pirt-GCaMP3 | Oxaliplatin | 10 | 9 | 19 |
| Supplementary Figure 2 | LTMR testing - imaging | Pirt-GCaMP4 | Oxaliplatin | 2 | 1 | 3 |
| | | | PNL | 3 | 3 | 6 |
| | | | P-CTX-2 | 4 | 6 | 10 |
| Supplementary Figure 6 | TTXr Na+ current recording | Nav1.8-Cre heterozygous tdTomato Pirt-GCaMP3 | TTX | | | 1 |
| | | Nav1.8-Cre homozygous tdTomato Pirt-GCaMP3 | | | | 1 |
| Supplementary Figure 6 | NaV1.8 KO - Behaviour | Nav1.8 WT | Vehicle | 3 | 3 | 6 |
| | | Nav1.8 WT | Oxaliplatin | 2 | 4 | 6 |
| | | Nav1.8 KO | | 4 | 4 | 8 |

## 3.3 Supplementary Table 3

| PCR PRODUCT | FORWARD PRIMER | REVERSE PRIMER |
|---|---|---|
| *Pirt-GCaMP3* | | |
| *Pirt WT (300 b.p.)* | TCCCCTCTACTGAGAGCCAG | GGCCCTATCATCCTGAGCAC |
| *GCaMP3 (400 b.p.)* | TCCCCTCTACTGAGAGCCAG | ATAGCTCTGACTGCGTGACC |
| *$Na_V$1.7 flox* | | |
| *$Na_V$1.7 WT (382 b.p.)* | CAGAGATTTCTGCATTAGAATTTGTTC | GCAAATCATAATTAATTCATGACACAG |
| *$Na_V$1.7 flox (527 b.p.)* | CAGAGATTTCTGCATTAGAATTTGTTC | GCAAATCATAATTAATTCATGACACAG or AGTCTTTGTGGCACACGTTACCTC |
| *$Na_V$1.7 KO (395 b.p.)* | CAGAGATTTCTGCATTAGAATTTGTTC | GTTCCTCTCTTTGAATGCTGGGCA |
| *Advillin-Cre* | | |
| *Advillin WT (480 b.p.)* | CCCTGTTCACTGTGAGTAGG | AGTATCTGGTAGGTGCTTCCAG |
| *Cre (180 b.p.)* | CCCTGTTCACTGTGAGTAGG | GCGATCCCTGAACATGTCCATC |
| *Rosa-flox-stop tdTomato (Ai9)* | | |
| *WT (297 b.p.)* | AAGGGAGCTGCAGTGGAGTA | CCGAAAATCTGTGGGAAGTC |
| *tdTomato (196 b.p.)* | CTGTTCCTGTACGGCATGG | GGCATTAAAGCAGCGTATCC |
| *Calb1-Cre* | | |
| *Calb1 WT (311 b.p.)* | AGAACATAATGGCCTTGTG | TACTGACTGGCCTAAGCATGG |
| *Cre (144 b.p.)* | AGAACATAATGGCCTTGTG | ACACCGGCCTTATTCCAAG |

| PCR PRODUCT | FORWARD PRIMER | REVERSE PRIMER |
|---|---|---|
| TrkB-CreERT2 | | |
| Ntrk2 WT (302 b.p.) | GACACGCACTCCGACTGACT | ACACCTGCCTGATTCCTGAG |
| CreERT2 (500 b.p.) | GCATGAAGTGCAAGAACGTG | ACACCTGCCTGATTCCTGAG |
| Trpv1-Cre | | |
| Trpv1 WT (490 b.p.) | TTCAGGGAGAAACTGGAAGAA | TAGTCCCAGCCATCCAAAAG |
| Cre (102 b.p.) | GCGGTCTGGCAGTAAAAACTATC | GTGAAACAGCATTGCTGTCACTT |
| $Na_V$1.8-Cre | | |
| $Na_V$1.8 WT (258 b.p.) | CAGTGGTCAGGCTGTCACCA | ACAGGCCTTCAAGTCCAACTG |
| Cre (346 b.p.) | CAGTGGTCAGGCTGTCACCA | AAATGTTGCTGGATAGTTTTTACTGCC |
| Tmem45b-Cre | | |
| Tmem45b-Cre (368 b.p.) | AGGCCAATGAGAAGTCCTGTGT | GGTATGCTCAGAAAACGCCTGG |
| CGRPα-CreERT2 | | |
| CreERT2 (900 b.p.) | TGCGGCGGATCCGAAAAGAA | TGCCAGGTTGGTCAGTAAGC |
| $Na_V$1.8 KO | | |
| $Na_V$1.8 WT (258 b.p.) | GAGTGATGCATATGATGTCAT | GCCTTCACTGTTGTTTACACCT |
| $Na_V$1.8 KO (346 b.p.) | GAGTGATGCATATGATGTCAT | GCAGCGCATCGCCTTCTATC |
| Rosa-flox-stop-DTA | | |
| WT (600 b.p.) | AAAGTCGCTCTGAGTTGTTAT | GGAGCGGGAGAAATGGATATG |
| DTA (250 b.p.) | AAAGTCGCTCTGAGTTGTTAT | GCGAAGAGTTTGTCCTCAACC |

## 3.4 Supplementary Table 4

| REAGENT | SOURCE | IDENTIFIER | DOSE | ADMIN. ROUTE |
|---|---|---|---|---|
| Oxaliplatin | Merck (Sigma) | O9512; CAS: 61825-94-3 | 80 µg in 40 µl of 5% glucose dH20 solution | Hindpaw intraplantar |
| Ciguatoxin-2 | Richard Lewis, University of Queensland | N/A | 100 nM in 20 µl of 1% BSA 0.5% methanol saline solution | Hindpaw intraplantar |
| Tamoxifen | Merck (Sigma) | T5648; CAS: 10540-29-1 | 1% in 200 µl of 15% ethanol 85% sunflower oil | Intraperitoneal |
| TTX | Merck (Sigma) | T8024; CAS: 4368-28-9 | 20 µM in 20 µl of saline | Hindpaw intraplantar |
| 4,9-anhydrousTTX | Tocris | 6159; CAS: 13072-89-4 | 20 µM in 20 µl of saline | Hindpaw intraplantar |
| Veratridine | Merck (Sigma) | V5754; CAS: 71-62-5 | 100 µM in 20 µl of saline | Hindpaw intraplantar |
| 4-aminopyridine | Merck (Sigma) | 275875; CAS: 504-24-5 | 10 mM in 20 µl of saline | Hindpaw intraplantar |
| α-dendrotoxin | Alomone labs | D-350; CAS: 74504-53-3 | 100 µM in 20 µl of saline | Hindpaw intraplantar |
| κ-dendrotoxin | Alomone labs | D-400; CAS: 119128-61-9 | 100 µM in 20 µl of saline | Hindpaw intraplantar |
| conotoxin kappaM-RIIIJ | Alomone labs | STC-660; CAS: N/A | 100 µM in 20 µl of saline | Hindpaw intraplantar |

## 3.5 Supplementary Table 5

| FIG. | NAME | VARIABLE (UNITS) | N | TEST | COMPARISON | STATISTIC | P | POST-HOC ANALYSIS | COMPARISON | P (ADJUSTED) |
|---|---|---|---|---|---|---|---|---|---|---|
| 1A | Cold Plate | Behaviours (#ipsi - #contra) | Veh: 8; Oxa: 9 | Repeated Measures 2-Way ANOVA | Interaction | F (1, 15) = 38.06 | <0.0001 | Sidak's multiple comparisons test | Vehicle - Basal vs 3 hours | 0.9987 |
| | | | | | Time | F (1, 15) = 37.25 | <0.0001 | | Oxaliplatin - Basal vs 3 hours | <0.0001 |
| | | | | | Treatment | F (1, 15) = 38.70 | <0.0001 | | | |
| 1A | Von Frey | Threshold (g) | Veh: 8; Oxa: 9 | Repeated Measures 2-Way ANOVA | Interaction | F (3, 30) = 13.12 | <0.0001 | Sidak's multiple comparisons test | Ipsi - Vehicle - Basal vs 3 hours | >0.9999 |
| | | | | | Time | F (1, 30) = 21.53 | <0.0001 | | Ipsi- Oxaliplatin - Basal vs 3 hours | <0.0001 |
| | | | | | Treatment | F (3, 30) = 9.535 | 0.0001 | | Contra - Vehicle - Basal vs 3 hours | 0.1908 |
| | | | | | | | | | Contra - Oxaliplatin - Basal vs 3 hours | 0.9978 |
| 1A | Hot Plate | Latency (s) | Veh: 8; Oxa: 9 | Repeated Measures 2-Way ANOVA | Interaction | F (1, 15) = 0.1999 | 0.6612 | Sidak's multiple comparisons test | Vehicle - Basal vs 3 hours | 0.9409 |
| | | | | | Time | F (1, 15) = 0.8208 | 0.3793 | | Oxaliplatin - Basal vs 3 hours | 0.5639 |
| | | | | | Treatment | F (1, 15) = 0.01760 | 0.8962 | | | |
| 1Ci | Silent cold-sensing neurons | Area (um2) | Veh: 82; Oxa: 179 | Kolmogorov-Smirnov | Veh. vs Oxa. | K-S D = 0.586 | <0.0001 | | | |
| 1Cii | Population | % responding cells | Veh: 383; Oxa: 542 | Chi-square | Veh. vs Oxa. | $\chi^2 = 69.88$ | <0.0001 | | | |
| 1D | Thresholds | Slope | Veh: 87; Oxa: 39 | Linear regression | Veh. vs Oxa. | F = 3.125 | 0.12 | | | |
| 1Ei | Heat/cold - ice water | Heat-responsive (%cold) | Veh: 51; Oxa: 81 | Chi-square | Veh. vs Oxa. | $\chi^2 = 0.05554$ | 0.8137 | | | |

| FIG. | NAME | VARIABLE (UNITS) | N | TEST | COMPARISON | STATISTIC | P | POST-HOC ANALYSIS | COMPARISON | P (ADJUSTED) |
|---|---|---|---|---|---|---|---|---|---|---|
| 1Ei | Heat/cold - acetone | Heat-responsive (%cold) | Veh: 58; Oxa: 145 | Chi-square | Veh. vs Oxa. | $\chi^2 = 0.4109$ | 0.5215 | | | |
| 1Eii | Mech./cold - ice water | Mechano-responsive (%cold) | Veh: 51; Oxa: 81 | Chi-square | Veh. vs Oxa. | $\chi^2 = 5.002$ | 0.0253 | | | |
| 1Eii | Mech./cold - acetone | Mechano-responsive (%cold) | Veh: 58; Oxa: 145 | Chi-square | Veh. vs Oxa. | $\chi^2 =11.93$ | 0.0006 | | | |
| 1Eiii | Mechano-cold | Area (um2) | Veh: 14; Oxa: 62 | Kolmogorov-Smirnov | Veh. vs Oxa. | K-S D = 0.3796 | 0.0014 | | | |
| 2A | Von Frey | Threshold (g) | Sham: 3; PNL: 6 | Repeated Measures 2-Way ANOVA | Interaction | F (2, 14) = 0.7224 | 0.5028 | Sidak's multiple comparisons test | Sham - Basal vs. 2 weeks | 0.6438 |
| | | | | | Time | F (2, 14) = 2.796 | 0.0952 | | Sham - Basal vs. 4 weeks | 0.9341 |
| | | | | | Surgery | F (1, 7) = 0.8268 | 0.3934 | | PNL - Basal vs. 2 weeks | 0.033 |
| | | | | | | | | | PNL - Basal vs. 4 weeks | 0.0529 |
| 2A | Acetone | Behaviours (s) | Sham: 3; PNL: 6 | Repeated Measures 2-Way ANOVA | Interaction | F (2, 14) = 4.447 | 0.032 | Sidak's multiple comparisons test | Sham - Basal vs. 2 weeks | 0.9915 |
| | | | | | Time | F (2, 14) = 0.4382 | 0.6537 | | Sham - Basal vs. 4 weeks | 0.5437 |
| | | | | | Surgery | F (1, 7) = 0.7745 | 0.408 | | PNL - Basal vs. 2 weeks | 0.9228 |
| | | | | | | | | | PNL - Basal vs. 4 weeks | 0.0316 |
| 2A | Hot Plate | Latency (s) | Sham: 3; PNL: 6 | Unpaired t-test (two-tailed) | Sham vs PNL - 4 weeks | t=0.8935 | 0.4013 | | | |
| 2Ci | Silent cold-sensing neurons | Area (um2) | Sham: 113; PNL: 109 | Kolmogorov-Smirnov | Sham vs PNL | K-S D = 0.3797 | <0.0001 | | | |

| FIG. | NAME | VARIABLE (UNITS) | N | TEST | COMPARISON | STATISTIC | P | POST-HOC ANALYSIS | COMPARISON | P (ADJUSTED) |
|---|---|---|---|---|---|---|---|---|---|---|
| 2Cii | Population | % responding cells | Sham: 373; PNL: 297 | Chi-square | Sham vs PNL | $\chi^2 = 40.70$ | <0.0001 | | | |
| 2D | Thresholds | Slope | Sham: 51; PNL: 40 | Linear regression | Sham vs PNL | F = 0.6035 | 0.4806 | | | |
| 2Ei | Heat/cold - ice water | Heat-responsive (%cold) | Sham: 64; PNL: 71 | Chi-square | Sham vs PNL | $\chi^2 = 0.03395$ | 0.8538 | | | |
| 2Ei | Heat/cold - acetone | Heat-responsive (%cold) | Sham: 95; PNL: 73 | Chi-square | Sham vs PNL | $\chi^2 = 2.327$ | 0.1272 | | | |
| 2Eii | Mech./cold - ice water | Mechano-responsive (%cold) | Sham: 64; PNL: 71 | Chi-square | Sham vs PNL | $\chi^2 = 6.909$ | 0.0086 | | | |
| 2Eii | Mech./cold - acetone | Mechano-responsive (%cold) | Sham: 95; PNL: 73 | Chi-square | Sham vs PNL | $\chi^2 = 9.506$ | 0.002 | | | |
| 3A | Acetone test | Behaviours (s) | Veh: 6; P-CTX-2: 6 | Repeated Measures 2-Way ANOVA | Interaction | F (1, 10) = 28.49 | 0.0003 | Sidak's multiple comparisons test | Vehicle - Basal vs 30 mins | 0.7175 |
| | | | | | Time | F (1, 10) = 41.00 | <0.0001 | | P-CTX-2 - Basal vs 30 mins | <0.0001 |
| | | | | | Treatment | F (1, 10) = 27.40 | 0.0004 | | | |
| 3A | Unilateral Cold Plate | Latency (s) | Veh: 6; P-CTX-2: 6 | Repeated Measures 2-Way ANOVA | Interaction | F (1, 10) = 7.418 | 0.0214 | Sidak's multiple comparisons test | Vehicle - Basal vs 30 mins | 0.8366 |
| | | | | | Time | F (1, 10) = 12.24 | 0.0057 | | P-CTX-2 - Basal vs 30 mins | 0.0027 |
| | | | | | Treatment | F (1, 10) = 1.760 | 0.2141 | | | |
| 3D | Silent cold-sensing neurons | Area (um2) | Pre: 91; Post: 206 | Kolmogorov-Smirnov | P-CTX-2 - Pre vs Post | K-S D = 0.4839 | <0.0001 | | | |

| FIG. | NAME | VARIABLE (UNITS) | N | TEST | COMPARISON | STATISTIC | P | POST-HOC ANALYSIS | COMPARISON | P (ADJUSTED) |
|---|---|---|---|---|---|---|---|---|---|---|
| 3Ei | Threshold change - Vehicle vs P-CTX-2 | Threshold change (change in degrees Celsius) | Veh: 35; P-CTX-2: 8 | Mann-Whitney U | Vehicle vs P-CTX-2 | U = 113.5 | 0.4043 | | | |
| 3Eii | Threshold - Basal v Silent | Threshold (degrees Celsius) | Basal: 62; Silent: 43 | Mann-Whitney U | Basal vs Silent | U = 1297 | 0.8093 | | | |
| 3Fi | Mech/cold - vehicle | Mechano-responsive (%cold) | Pre: 36; Post: 43 | Chi-square | Vehicle - Pre vs Post | $\chi^2 = 0.05138$ | 0.8207 | | | |
| 3Fi | Mech/cold - P-CTX-2 | Mechano-responsive (%cold) | Pre: 69; Post: 174 | Chi-square | P-CTX-2 - Pre vs Post | $\chi^2 = 4.069$ | 0.0437 | | | |
| 3Fii | Heat/cold - vehicle | Heat-responsive (%cold) | Pre: 36; Post: 43 | Chi-square | Vehicle - Pre vs Post | $\chi^2 = 0.4481$ | 0.5032 | | | |
| 3Fii | Heat/cold - P-CTX-2 | Heat-responsive (%cold) | Pre: 69; Post: 174 | Chi-square | P-CTX-2 - Pre vs Post | $\chi^2 = 3.627$ | 0.0568 | | | |
| 3Fiii | Silent - mech/cold | Mechano-responsive (%cold) | Silent: 127 | Chi-square | Basal vs Silent | $\chi^2 = 0.000$ | >0.9999 | | | |
| 3Fiii | Silent - heat/cold | Heat-responsive (%cold) | Silent: 127 | Chi-square | Basal vs Silent | $\chi^2 = 6.281$ | 0.0122 | | | |
| 5Bii | In vivo imaging | Area (um2) | NaV1.8-Cre WT: 108; NaV1.8-Cre DTA: 46 | Kolmogorov-Smirnov | Oxaliplatin - WT vs DTA | K-S D = 0.3937 | <0.0001 | | | |

| FIG. | NAME | VARIABLE (UNITS) | N | TEST | COMPARISON | STATISTIC | P | POST-HOC ANALYSIS | COMPARISON | P (ADJUSTED) |
|---|---|---|---|---|---|---|---|---|---|---|
| 5C | Behaviour | Behaviours (#ipsi - #contra) | WT DTA: 10; NaV1.8-Cre DTA: 8 | Mann-Whitney U | Oxaliplatin - WT vs DTA | U = 11 | 0.0085 | | | |
| 6Aii | Homozygous NaV1.8-Cre | Area (um2) | Het: 66; Homo: 42 | Kolmogorov-Smirnov | Oxaliplatin - Het vs Homo | K-S D = 0.2121 | 0.1984 | | | |
| 6Aiii | Homozygous NaV1.8-Cre | tdTomato-positive (%silent cold) | Het: 36; Homo: 28 | Chi-square | Oxaliplatin - Het vs Homo | $\chi^2$ = 0.08044 | 0.7767 | | | |
| 6B | NaV1.7 KO | Area (um2) | WT: 51; NaV1.7 KO: 18 | Kolmogorov-Smirnov | Oxaliplatin - WT vs NaV1.7 KO | K-S D = 0.2484 | 0.3846 | | | |
| 6Cii | Blockers - small basal cells | Response magnitude (dF/F0) | | Kruskall Wallis | | K-W Statistic = 56.1 | <0.0001 | Dunn's multiple comparisons test | Saline - basal vs treatment | >0.9999 |
| | | | | | | | | | TTX - basal vs treatment | <0.0001 |
| | | | | | | | | | anhyTTX - basal vs treatment | >0.9999 |
| 6Cii | Blockers - large silent cells | Response magnitude (dF/F0) | | Kruskall Wallis | | K-W Statistic = 21.04 | 0.0008 | Dunn's multiple comparisons test | Saline - basal vs treatment | >0.9999 |
| | | | | | | | | | TTX - basal vs treatment | 0.0022 |
| | | | | | | | | | anhyTTX - basal vs treatment | 0.0382 |
| 6Dii | Veratridine - Cell Area | Cell Area (um2) | Naïve: 39; Vera: 31 | Mann Whitney test | Naïve vs Vera | U = 603 | 0.9883 | | | |
| 6Div | Veratridine - Response magnitude | Response magnitude (dF/F0) | All: 53 | Wilcoxon matched-pairs signed rank test | Naïve vs Vera (Pre vs Post) | W = -525 | 0.0195 | | | |

| FIG. | NAME | VARIABLE (UNITS) | N | TEST | COMPARISON | STATISTIC | P | POST-HOC ANALYSIS | COMPARISON | P (ADJUSTED) |
|---|---|---|---|---|---|---|---|---|---|---|
| 7D | Neuron sizes of unmasked population | Cell Area (um2) | Saline - Basal: 30; Silent: 12 | Kruskall Wallis | | K-W Statistic = 117.5 | <0.0001 | Dunn's multiple comparisons test | Saline - Basal vs Silent | 0.365 |
| | | | 4-AP - Basal: 24; Silent: 33 | | | | | | 4-AP - Basal vs Silent | <0.0001 |
| | | | 4-AP + Oxaliplatin - Basal: 49; Silent: 46 | | | | | | 4-AP + Oxa - Basal vs Silent | 0.0861 |
| | | | α-DTx - Basal: 56; Silent: 45 | | | | | | α-DTx - Basal vs Silent | 0.001 |
| | | | k-DTx - Basal: 29; Silent: 45 | | | | | | k-DTx - Basal vs Silent | 0.0671 |
| | | | RIIIJ - Basal: 10; Silent: 4 | | | | | | RIIIJ - Basal vs Silent | >0.9999 |
| 7E | Saline - Polymodality | Mechanical (%cold) | Saline - Naive: 30; Treatment: 32 | Chi-square | Oxaliplatin - Het vs Homo | $\chi^2 = 0.2422$ | 0.6226 | | | |
| 7E | 4-AP - Polymodality | Mechanical (%cold) | 4-AP - Naive: 24; Treatment: 48 | Chi-square | Oxaliplatin - Het vs Homo | $\chi^2 = 9.474$ | 0.0021 | | | |
| 7E | 4-AP + Oxa - Polymodality | Mechanical (%cold) | 4-AP + Oxa - Oxa: 49; Treatment: 78 | Chi-square | Oxaliplatin - Het vs Homo | $\chi^2 = 2.700$ | 0.1003 | | | |
| 7E | α-DTx - Polymodality | Mechanical (%cold) | α-DTx - Naive: 30; Treatment: 32 | Chi-square | Oxaliplatin - Het vs Homo | $\chi^2 = 5.671$ | 0.0172 | | | |
| 7E | k-DTx - Polymodality | Mechanical (%cold) | k-DTx - Naive: 29; Treatment: 47 | Chi-square | Oxaliplatin - Het vs Homo | $\chi^2 = 0.03081$ | 0.8607 | | | |
| 7E | RIIIJ - Polymodality | Mechanical (%cold) | RIIIJ - Naive: 10; Treatment: 11 | Chi-square | Oxaliplatin - Het vs Homo | $\chi^2 = 1.155$ | 0.2825 | | | |

| FIG. | NAME | VARIABLE (UNITS) | N | TEST | COMPARISON | STATISTIC | P | POST-HOC ANALYSIS | COMPARISON | P (ADJUSTED) |
|---|---|---|---|---|---|---|---|---|---|---|
| 7D | Response magnitude | Response magnitude (dF/d0) | Saline: 20 | Kruskall Wallis | | K-W Statistic = 24.64 | 0.0103 | Dunn's multiple comparisons test | Saline - Naïve vs Treatment | >0.9999 |
| | | | 4-AP: 15 | | | | | | 4-AP - Naïve vs Treatment | >0.9999 |
| | | | 4-AP + Oxa: 32 | | | | | | 4-AP + Oxa - Oxa vs Treatment | 0.2811 |
| | | | α-DTx: 35 | | | | | | α-DTx - Naïve vs Treatment | >0.9999 |
| | | | k-DTx: 28 | | | | | | k-DTx - Naïve vs Treatment | >0.9999 |
| | | | RIIIJ: 7 | | | | | | RIIIJ - Naïve vs Treatment | >0.9999 |

## 3.6 Supplementary Table 6

| SUP. FIG. | NAME | VARIABLE (UNITS) | N | TEST | COMPARISON | STATISTIC | P | POST-HOC ANALYSIS | COMPARISON | P (ADJUSTED) |
|---|---|---|---|---|---|---|---|---|---|---|
| 1A | Cold Plate | Behaviours (#ipsi - #contra) | Male Veh: 5; Male Oxa: 5; Female Veh: 3; Female Oxa: 4 | Repeated Measures 2-Way ANOVA | Interaction | F (3, 13) = 21.32 | <0.0001 | Sidak's multiple comparisons test | Male - Vehicle - Basal vs 3 hours | >0.9999 |
| | | | | | Time | F (1, 13) = 54.54 | <0.0001 | | Male - Oxaliplatin - Basal vs 3 hours | 0.0002 |
| | | | | | Treatment | F (3, 13) = 26.08 | <0.0001 | | Female - Vehicle - Basal vs 3 hours | 0.9993 |
| | | | | | | | | | Female - Oxaliplatin - Basal vs 3 hours | <0.0001 |
| 1A | Von Frey | Threshold (g) | Male Veh: 5; Male Oxa: 5; Female Veh: 3; Female Oxa: 4 | Repeated Measures 2-Way ANOVA | Interaction | F (3, 13) = 6.359 | 0.0069 | Sidak's multiple comparisons test | Male - Vehicle - Basal vs 3 hours | 0.9984 |
| | | | | | Time | F (1, 13) = 17.82 | 0.001 | | Male - Oxaliplatin - Basal vs 3 hours | 0.0009 |
| | | | | | Treatment | F (3, 13) = 4.723 | 0.0193 | | Female - Vehicle - Basal vs 3 hours | 0.9987 |
| | | | | | | | | | Female - Oxaliplatin - Basal vs 3 hours | 0.007 |
| 1A | Hot Plate | Latency (s) | Male Veh: 5; Male Oxa: 5; Female Veh: 3; Female Oxa: 4 | Repeated Measures 2-Way ANOVA | Interaction | F (3, 13) = 0.1900 | 0.9013 | Sidak's multiple comparisons test | Male - Vehicle - Basal vs 3 hours | 0.9791 |
| | | | | | Time | F (1, 13) = 0.5241 | 0.4819 | | Male - Oxaliplatin - Basal vs 3 hours | 0.8116 |
| | | | | | Treatment | F (3, 13) = 0.3497 | 0.7901 | | Female - Vehicle - Basal vs 3 hours | 0.9997 |
| | | | | | | | | | Female - Oxaliplatin - Basal vs 3 hours | 0.9974 |
| 1B | Ice water - area | Area (um2) | Veh: 51; Oxa: 81 | Kolmogorov-Smirnov | Veh. vs Oxa. | K-S D = 0.5694 | 0.0001 | | | |
| 1B | Acetone - area | Area (um2) | Veh: 51; Oxa: 145 | Kolmogorov-Smirnov | Veh. vs Oxa. | K-S D = 0.6483 | 0.0001 | | | |
| 1B | Mechanical - area | Area (um2) | Veh: 136; Oxa: 193 | Kolmogorov-Smirnov | Veh. vs Oxa. | K-S D = 0.1127 | 0.263 | | | |
| 1B | Heat - area | Area (um2) | Veh: 211; Oxa: 301 | Kolmogorov-Smirnov | Veh. vs Oxa. | K-S D = 0.1308 | 0.0287 | | | |
| 1C | Acetone test | Behaviours (s) | Oxa: 7 | Repeated Measures 2-Way ANOVA | Interaction | F (1, 12) = 33.89 | <0.0001 | Sidak's multiple comparisons test | Ipsi - Oxaliplatin - Basal vs 3 hours | <0.0001 |
| | | | | | Paw | F (1, 12) = 34.58 | <0.0001 | | Contra- Oxaliplatin - Basal vs 3 hours | 0.999 |
| | | | | | Time | F (1, 12) = 36.01 | <0.0001 | | | |

| SUP. FIG. | NAME | VARIABLE (UNITS) | N | TEST | COMPARISON | STATISTIC | P | POST-HOC ANALYSIS | COMPARISON | P (ADJUSTED) |
|---|---|---|---|---|---|---|---|---|---|---|
| 1D | Cold response magnitude - Ice water | Response magnitude (dF/F0) | Veh: 51; Basal: 40; Silent: 41 | Kruskall Wallis | | K-W statistic = 3.267 | 0.1952 | Dunn's multiple comparisons test | Vehicle vs Basal | >0.9999 |
| | | | | | | | | | Vehicle vs Silent | 0.1869 |
| 1D | Cold response magnitude - acetone | Response magnitude (dF/F0) | Veh: 58; Basal: 57; Silent: 88 | Kruskall Wallis | | K-W statistic = 8.861 | 0.0119 | Dunn's multiple comparisons test | Vehicle vs Basal | 0.4398 |
| | | | | | | | | | Vehicle vs Silent | 0.2188 |
| 1E | Modality populations - mech | Pinch-responsive (%cells) | Veh: 383; Oxa: 542 | Chi-square | Veh. vs Oxa. | $\chi^2 = 0.0009737$ | 0.9751 | | | |
| 1E | Modality populations - heat | Heat-responsive (%cells) | Veh: 383; Oxa: 542 | Chi-square | Veh. vs Oxa. | $\chi^2 = 0.01787$ | 0.8936 | | | |
| 1F | Mechanical | Response magnitude (dF/F0) | Veh: 136; Oxa: 193 | Mann-Whitney U | Veh. vs Oxa. | U = 13001 | 0.8854 | | | |
| 1F | Heat | Response magnitude (dF/F0) | Veh: 211; Oxa: 301 | Mann-Whitney U | Veh. vs Oxa. | U = 26386 | 0.0011 | | | |
| 1G | Polymodalit y | Mechanoheat (%mechanical) | Veh: 136; Oxa: 193 | Chi-square | Veh. vs Oxa. | $\chi^2 = 6.397$ | 0.0114 | | | |
| 1H | Ice water | Area (um2) | Veh: 8; Oxa: 27 | Kolmogorov-Smirnov | Veh. vs Oxa. | K-S D = 0.3796 | 0.336 | | | |
| 1H | Acetone | Area (um2) | Veh: 7; Oxa: 53 | Kolmogorov-Smirnov | Veh. vs Oxa. | K-S D = 0.7817 | 0.001 | | | |
| 3A | Cold allodynia | Latency (s) | Sham: 3; PNL: 6 | Unpaired t-test (two-tailed) | Sham vs PNL - 4 weeks | t=2.524 | 0.0396 | | | |
| 3B | Von Frey | Threshold (g) | Male: 3; Female: 3 | Repeated Measures 2-Way ANOVA | Interaction | F (2, 8) = 0.03499 | 0.9658 | Sidak's multiple comparisons test | Male - Basal vs. 2 weeks | 0.2672 |
| | | | | | Time | F (2, 8) = 3.129 | 0.0991 | | Male - Basal vs. 4 weeks | 0.2649 |
| | | | | | Sex | F (1, 4) = 0.3816 | 0.5702 | | Female - Basal vs. 2 weeks | 0.283 |
| | | | | | | | | | Female - Basal vs. 4 weeks | 0.4153 |

| SUP. FIG. | NAME | VARIABLE (UNITS) | N | TEST | COMPARISON | STATISTIC | P | POST-HOC ANALYSIS | COMPARISON | P (ADJUSTED) |
|---|---|---|---|---|---|---|---|---|---|---|
| 3B | Acetone | Behaviours (s) | Male: 3; Female: 3 | Repeated Measures 2-Way ANOVA | Interaction | F (2, 8) = 0.09377 | 0.9115 | Sidak's multiple comparisons test | Male - Basal vs. 2 weeks | 0.9961 |
| | | | | | Time | F (2, 8) = 4.416 | 0.051 | | Male - Basal vs. 4 weeks | 0.1983 |
| | | | | | Sex | F (1, 4) = 11.44 | 0.0277 | | Female - Basal vs. 2 weeks | 0.8483 |
| | | | | | | | | | Female - Basal vs. 4 weeks | 0.2884 |
| 3B | Unilateral Cold Plate | Latency (s) | Male: 3; Female: 3 | Unpaired t-test (two-tailed) | Male vs Female - 4 weeks | t=0.5203 | 0.6303 | | | |
| 3B | Hot Plate | Latency (s) | Male: 3; Female: 3 | Unpaired t-test (two-tailed) | Male vs Female - 4 weks | t=1.783 | 0.1492 | | | |
| 3C | Ice water - area | Area (um2) | Sham: 64; PNL: 71 | Kolmogorov-Smirnov | Sham vs PNL | K-S D = 0.3371 | 0.001 | | | |
| 3C | Acetone - area | Area (um2) | Sham: 95; PNL: 73 | Kolmogorov-Smirnov | Sham vs PNL | K-S D = 0.4016 | <0.0001 | | | |
| 3C | Mechanical - area | Area (um2) | Sham: 105; PNL: 114 | Kolmogorov-Smirnov | Sham vs PNL | K-S D = 0.09198 | 0.09198 | | | |
| 3C | Heat - area | Area (um2) | Sham: 174; PNL: 118 | Kolmogorov-Smirnov | Sham vs PNL | K-S D = 0.3456 | 0.3456 | | | |
| 3D | Cold response magnitude - Ice water | Response magnitude (dF/F0) | Sham: 64; Basal: 46; Silent: 25 | Kruskall Wallis | | K-W Statistic = 5.496 | 0.0641 | Dunn's multiple comparisons test | Sham vs Basal | >0.9999 |
| | | | | | | | | | Sham vs Silent | 0.045 |
| 3D | Cold response magnitude - acetone | Response magnitude (dF/F0) | Sham: 95; Basal: 42; Silent: 31 | Kruskall Wallis | | K-W Statistic = 15.82 | 0.0004 | Dunn's multiple comparisons test | Sham vs Basal | 0.0166 |
| | | | | | | | | | Sham vs Silent | 0.0655 |
| 3F | Modality populations - mech | Pinch-responsive (%cells) | Sham: 373; PNL: 297 | Chi-square | Sham vs PNL | $\chi^2$ = 7.870 | 0.005 | | | |
| 3F | Modality populations - heat | Heat-responsive (%cells) | Sham: 373; PNL: 297 | Chi-square | Sham vs PNL | $\chi^2$ = 3.218 | 0.0728 | | | |
| 3G | Mechanical | Response magnitude (dF/F0) | Sham: 105; PNL: 114 | Mann-Whitney U | Sham vs PNL | U = 3788 | <0.0001 | | | |
| 3G | Heat | Response magnitude (dF/F0) | Sham: 174; PNL: 118 | Mann-Whitney U | Sham vs PNL | U = 9146 | 0.1139 | | | |
| 3H | Polymodalit y | Mechanoheat (%mechanical) | Sham: 105; PNL: 114 | Chi-square | Sham vs PNL | $\chi^2$ = 1.054 | 0.3047 | | | |

| SUP. FIG. | NAME | VARIABLE (UNITS) | N | TEST | COMPARISON | STATISTIC | P | POST-HOC ANALYSIS | COMPARISON | P (ADJUSTED) |
|---|---|---|---|---|---|---|---|---|---|---|
| 4A | Acetone | Behaviours (s) | Male Veh: 3; Male P-CTX-2: 3; Female Veh: 3; Female P-CTX-2: 3 | Repeated Measures 2-Way ANOVA | Interaction | F (3, 8) = 15.51 | 0.0011 | Sidak's multiple comparisons test | Male - Vehicle - Basal vs 30 mins | 0.6169 |
| | | | | | Time | F (1, 8) = 58.10 | <0.0001 | | Male - P-CTX-2 - Basal vs 30 mins | 0.0026 |
| | | | | | Treatment | F (3, 8) = 13.88 | 0.0015 | | Female - Vehicle - Basal vs 30 mins | >0.9999 |
| | | | | | | | | | Female - P-CTX-2 - Basal vs 30 mins | 0.0001 |
| 4A | Unilateral Cold Plate | Latency (s) | Male Veh: 3; Male P-CTX-2: 3; Female Veh: 3; Female P-CTX-2: 3 | Repeated Measures 2-Way ANOVA | Interaction | F (3, 8) = 3.138 | 0.087 | Sidak's multiple comparisons test | Male - Vehicle - Basal vs 30 mins | 0.9737 |
| | | | | | Time | F (1, 8) = 12.24 | 0.0081 | | Male - P-CTX-2 - Basal vs 30 mins | 0.0945 |
| | | | | | Treatment | F (3, 8) = 0.6605 | 0.5991 | | Female - Vehicle - Basal vs 30 mins | 0.6332 |
| | | | | | | | | | Female - P-CTX-2 - Basal vs 30 mins | 0.034 |
| 4B | Ice-water Area | Area (um2) | Vehicle Pre: 36; Vehicle Post: 43; P-CTX-2 Pre: 69; P-CTX-2 Post: 174 | Kruskall Wallis | | K-W Statistic = 70.53 | <0.0001 | Dunn's multiple comparisons test | Vehicle - Pre vs Post | >0.9999 |
| | | | | | | | | | P-CTX-2 - Pre vs Post | <0.0001 |
| 4B | Acetone Area | Area (um2) | Vehicle Pre: 33; Vehicle Post: 39; P-CTX-2 Pre: 72; P-CTX-2 Post: 105 | Kruskall Wallis | | K-W Statistic = 15.65 | 0.0013 | Dunn's multiple comparisons test | Vehicle - Pre vs Post | >0.9999 |
| | | | | | | | | | P-CTX-2 - Pre vs Post | 0.0002 |
| 4B | Mechanical Area | Area (um2) | Vehicle Pre: 57; Vehicle Post: 48; P-CTX-2 Pre: 115; P-CTX-2 Post: 131 | Kruskall Wallis | | K-W Statistic = 9.574 | 0.0226 | Dunn's multiple comparisons test | Vehicle - Pre vs Post | >0.9999 |
| | | | | | | | | | P-CTX-2 - Pre vs Post | 0.0205 |
| 4B | Heat Area | Area (um2) | Vehicle Pre: 59; Vehicle Post: 77; P-CTX-2 Pre: 211; P-CTX-2 Post: 241 | Kruskall Wallis | | K-W Statistic = 5.193 | 0.1582 | Dunn's multiple comparisons test | Vehicle - Pre vs Post | >0.9999 |
| | | | | | | | | | P-CTX-2 - Pre vs Post | >0.9999 |
| 4C | Ice-water response magnitude | Response magnitude (dF/F0) | Vehicle: 36; P-CTX-2: 69 | Kruskall Wallis | | K-W Statistic = 17.02 | 0.0007 | Dunn's multiple comparisons test | Vehicle - Pre vs Post | 0.7016 |
| | | | | | | | | | P-CTX-2 - Pre vs Post | 0.0001 |
| 4C | Acetone response magnitude | Response magnitude (dF/F0) | Vehicle: 33; P-CTX-2: 72 | Kruskall Wallis | | K-W Statistic = 26.86 | <0.0001 | Dunn's multiple comparisons test | Vehicle - Pre vs Post | 0.1829 |
| | | | | | | | | | P-CTX-2 - Pre vs Post | <0.0001 |

| SUP. FIG. | NAME | VARIABLE (UNITS) | N | TEST | COMPARISON | STATISTIC | P | POST-HOC ANALYSIS | COMPARISON | P (ADJUSTED) |
|---|---|---|---|---|---|---|---|---|---|---|
| 4D | Silent - Ice-water | Response magnitude (dF/F0) | Basal: 105; Silent: 127 | Mann-Whitney U | Basal vs Silent | U = 5627 | 0.041 | | | |
| 4D | Silent - Acetone | Response magnitude (dF/F0) | Basal: 105; Silent: 60 | Mann-Whitney U | Basal vs Silent | U =3119 | 0.918 | | | |
| 4E | Mechanical response magnitude | Response magnitude (dF/F0) | Vehicle: 75; P-CTX-2: 190 | Kruskall Wallis | | K-W Statistic = 9.543 | 0.0229 | Dunn's multiple comparisons test | Vehicle - Pre vs Post<br>P-CTX-2 - Pre vs Post | 0.0191<br>0.6558 |
| 4E | Heat response magnitude | Response magnitude (dF/F0) | Vehicle: 91; P-CTX-2: 321 | Kruskall Wallis | | K-W Statistic = 6.168 | 0.1037 | Dunn's multiple comparisons test | Vehicle - Pre vs Post<br>P-CTX-2 - Pre vs Post | 0.1753<br>0.7272 |
| 4F | Polymodality - Mechanoheat - Vehicle | Mechanoheat (%mechanical) | Pre: 57; Post: 48 | Chi-square | Pre vs Post | $\chi^2$ = 0.8443 | 0.3582 | | | |
| 4F | Polymodality - Mechanoheat - P-CTX-2 | Mechanoheat (%mechanical) | Pre: 115; Post: 131 | Chi-square | Pre vs Post | $\chi^2$ = 6.714 | 0.0096 | | | |
| 4Gi | Mech/acetone - vehicle | Mechano-responsive (%acetone) | Pre: 33; Post: 39 | Chi-square | Vehicle - Pre vs Post | $\chi^2$ = 2.431 | 0.1189 | | | |
| 4Gi | Mech/acetone - P-CTX-2 | Mechano-responsive (%acetone) | Pre: 72; Post: 105 | Chi-square | P-CTX-2 - Pre vs Post | $\chi^2$ = 3.720 | 0.0538 | | | |
| 4Gii | Heat/acetone - vehicle | Heat-responsive (%acetone) | Pre: 33; Post: 39 | Chi-square | Vehicle - Pre vs Post | $\chi^2$ = 1.741 | 0.1871 | | | |
| 4Gii | Heat/acetone - P-CTX-2 | Heat-responsive (%acetone) | Pre: 72; Post: 105 | Chi-square | P-CTX-2 - Pre vs Post | $\chi^2$ = 0.1109 | 0.7391 | | | |
| 4Giii | Silent - mech/acetone | Mechano-responsive (%acetone) | Silent: 60 | Chi-square | Basal vs Silent | $\chi^2$ = 0.000 | >0.9999 | | | |
| 4Giii | Silent - heat/acetone | Heat-responsive (%acetone) | Silent: 60 | Chi-square | Basal vs Silent | $\chi^2$ = 4.138 | 0.0419 | | | |
| 6A | TTXr current | Current density (pA/pF) | Het: 5; Homo: 5 | Mann-Whitney U | Het vs Homo | U = 0 | 0.0079 | | | |
| 6B | NaV1.8 KO - Oxaliplatin | Behaviours (#ipsi - #contra) | WT Veh: 6; WT Oxa: 6; NaV1.8 KO Oxa: 8 | Kruskall Wallis | | K-W Statistic = 12.08 | 0.0004 | Dunn's multiple comparisons test | WT Vehicle vs WT Oxa<br>WT Vehicle vs NaV1.8 KO Oxa<br>WT Oxa vs NaV1.8 Oxa | 0.0126<br>0.0042<br>>0.9999 |

# 4 Supplementary References


Akopian AN, Souslova V, England S, Okuse K, Ogata N, Ure J, et al. The tetrodotoxin-resistant sodium channel SNS has a specialized function in pain pathways. Nat Neurosci 1999; 2: 541–8.

Allchorne AJ, Broom DC, Woolf CJ. Detection of cold pain, cold allodynia and cold hyperalgesia in freely behaving rats. Mol Pain 2005; 1

Cavanaugh DJ, Chesler AT, Jackson AC, Sigal YM, Yamanaka H, Grant R, et al. Trpv1 Reporter Mice Reveal Highly Restricted Brain Distribution and Functional Expression in Arteriolar Smooth Muscle Cells. J Neurosci 2011; 31: 5067–77.

Chaplan SR, Bach FW, Pogrel JW, Chung JM, Yaksh TL. Quantitative assessment of tactile allodynia in the rat paw. J Neurosci Methods 1994; 53: 55–63.

Deuis JR, Zimmermann K, Romanovsky AA, Possani LD, Cabot PJ, Lewis RJ, et al. An animal model of oxaliplatin-induced cold allodynia reveals a crucial role for Nav1.6 in peripheral pain pathways. Pain 2013; 154: 1749–57.

Ivanova A, Signore M, Caro N, Greene NDE, Copp AJ, Martinez-Barbera JP. In vivo genetic ablation by Cre-mediated expression of diphtheria toxin fragment A. Genesis 2005; 43: 129–35.

Kim YS, Chu Y, Han L, Li M, Li Z, Lavinka PC, et al. Central terminal sensitization of TRPV1 by descending serotonergic facilitation modulates chronic pain. Neuron 2014; 81: 873–87.

Madisen L, Zwingman TA, Sunkin SM, Oh SW, Zariwala HA, Gu H, et al. A robust and high-throughput Cre reporting and characterization system for the whole mouse brain. Nat Neurosci 2010; 13: 133–40.

Nassar MA, Stirling LC, Forlani G, Baker MD, Matthews EA, Dickenson AH, et al. Nociceptor-specific gene deletion reveals a major role for Nav1.7 (PN1) in acute and inflammatory pain. Proc Natl Acad Sci U S A 2004; 101: 12706–11.

Nigro MJ, Hashikawa-Yamasaki Y, Rudy B. Diversity and connectivity of layer 5 somatostatin-expressing interneurons in the mouse barrel cortex. J Neurosci 2018; 38: 1622–33.

Rutlin M, Ho C-Y, Abraira VE, Cassidy C, Bai L, Woodbury CJ, et al. The cellular and molecular basis of direction selectivity of Aδ-LTMRs. Cell 2014; 159: 1640–51.

Song H, Yao E, Lin C, Gacayan R, Chen MH, Chuang PT. Functional characterization of pulmonary neuroendocrine cells in lung development, injury,and tumorigenesis. Proc Natl Acad Sci U S A 2012; 109: 17531–6.

Woolfe G, MacDonald AD. The evaluation of the analgesic action of pethidine hydrochloride (Demerol). J Pharmacol Exp Ther 1944; 80

Yoon C, Young Wook Y, Heung Sik N, Sun Ho K, Jin Mo C. Behavioral signs of ongoing pain and cold allodynia in a rat model of neuropathic pain. Pain 1994; 59: 369–76.

Zhou X, Wang L, Hasegawa H, Amin P, Han BX, Kaneko S, et al. Deletion of PIK3C3/Vps34 in sensory neurons causes rapid neurodegeneration by disrupting the

endosomal but not the autophagic pathway. Proc Natl Acad Sci U S A 2010; 107: 9424–9.
